# Interaction of unphosphorylated PtsN with the K^+^/H^+^ antiporter YcgO inhibits its activity in *Escherichia coli*

**DOI:** 10.1016/j.jbc.2024.108153

**Published:** 2024-12-30

**Authors:** Yogesh Patidar, Arunabh Athreya, Ravish Sharma, Aravind Penmatsa, Abhijit A. Sardesai

**Affiliations:** 1 Laboratory of Molecular Microbiology and Genetics, BRIC-Centre for DNA Fingerprinting and Diagnostics, Hyderabad, India; 2 Graduate Studies Regional Centre for Biotechnology, Faridabad, India; 3 Molecular Biophysics Unit, Indian Institute of Science, Bangalore, India

**Keywords:** potassium transport, protein-protein interaction, membrane protein, bacterial genetics, bacterial metabolism, potassium/proton antiport, phosphorelay, *Escherichia coli* (*E. coli*), unphosphorylated-PtsN

## Abstract

Genetic studies in *Escherichia coli* have implicated the unphosphorylated version of PtsN (unphospho-PtsN), the terminal phospho-acceptor of the PtsP-PtsO-PtsN phosphorelay, as a negative regulator of potassium (K^+^) efflux mediated by YcgO. YcgO is a protein belonging to the CPA1 family of monovalent cation/proton antiporters. Here we show that *in vivo*, YcgO comprises an approximately 383 amino acid N-terminal transmembrane domain and a 195 amino acid C-terminal cytoplasmic region (CTR). Copurification studies show that unphospho-PtsN specifically interacts with YcgO, and phosphorylation of PtsN leads to marked attenuation of the interaction. Genetic and biochemical analyses of a class of mutations in YcgO that lead to constitutive activation of YcgO identify the CTR as the site of interaction between unphospho-PtsN and YcgO and indicate that the putative CorC domain in the CTR may serve as the site of interaction. Our studies are supportive of a model which postulates that the unphospho–PtsN:CorC interaction may inhibit the activation of YcgO by a putative RCK domain in the CTR, leading to the inhibition of the K^+^/H^+^ antiport activity of YcgO.

In *Escherichia coli* and in closely related members of Enterobacteriaceae, under laboratory growth conditions, some solute transport processes are naturally rendered silent. Distinct mechanisms maintain the transporter in the silent state. Thus, transport-related growth phenotypes and activities are seen only upon inactivation of the silencing mechanisms. For example, genes for both uptake and catabolism of environmental β-glucosides arbutin and salicin are silent in most strains of *E. coli* K-12, a phenomenon attributed to negative regulatory elements in the *cis* regulatory region of the *bgl* operon (1, 2). Uptake and catabolism of β-glucosides are a consequence of mutations that alleviate this promoter silencing (3). Functional substitution of PutP-mediated L-proline uptake in *Salmonella enterica* requires the unveiling of a cryptic L-proline importer ProY. This is attained either by a mutation in the *proZ* gene that unmasks ProY activity or by the overexpression of ProY (4).

KefG/B and KefF/C, potassium (K^+^) efflux (Kef) systems, perform efflux of K^+^ with H^+^ influx in antiport (5, 6), and they are also rendered silent, held in an inactive state by glutathione (7). KefB and KefC constitute the ion-conducting components, whereas full activity KefC-mediated K^+^ efflux requires KefF (8). KefG is thought to play a similar regulatory role for K^+^ efflux mediated by KefB. Conjugates of electrophiles with glutathione lead to the activation of K^+^ efflux and the accompanying acidification of the cytoplasm *via* H^+^ entry mitigates electrophile toxicity (9). The physiological impact of Kef-mediated K^+^ efflux is realized under conditions of exposure to the electrophile, methylglyoxal which also can form *in vivo* under certain metabolic conditions *via* the activity of MG synthase (10, 11). Unlike the aforementioned examples of cryptic β-glucoside and L-proline transport, the Kef system can be rendered cryptic following the decline of electrophile stress. For the Kef systems, the physiological need for the evolutionary retention of their silent nature can be rationalized on the basis that Kef activation may enable a rapid and a reversible response to electrophile stress (12). However, while the need for β-glucoside and L-proline uptake is easy to come to terms with, their evolutionary retention in a naturally silent state appears enigmatic.

Previously, we have described a physiological link between the phosphoenolpyruvate-dependent phosphorelay PtsP-PtsO-PtsN (13, 14) and cellular K^+^ metabolism (15). We showed that the growth inhibition of a mutant lacking PtsN, the terminal phospho-acceptor protein of the PtsP-PtsO-PtsN phosphorelay, in media with extracellular K^+^ concentrations ([K^+^]_e_s) greater than 20 mM, is associated with the unveiling of K^+^ efflux mediated by an inner membrane protein YcgO, leading to K^+^ limitation *in vivo* (15). Repression and inhibition of the activity of the inducible Kdp K^+^ uptake transporter (16, 17) by external K^+^ (18, 19, 20) is thought to be a factor in the maintenance of K^+^ limitation in the Δ*ptsN* mutant (15). Growth of the Δ*ptsN* mutant in a low [K^+^]_e_ medium occurs presumably owing to Kdp-mediated K^+^ uptake that counteracts YcgO-mediated K^+^ limitation. YcgO is a member of the monovalent cation/proton antiporters (CPA1) family (21). K^+^ limitation in the Δ*ptsN* mutant was alleviated either by the absence of YcgO or by the overexpression of a K^+^ uptake protein like Kup (15, 22). Circumstantial evidence from our study (15) along with direct evidence from another group (23, 24) has implicated the unphosphorylated version of PtsN, unphospho-PtsN as a negative regulator of YcgO activity. Thus, in *E. coli*, unphospho-PtsN maintains YcgO in a silent state.

In this study, we have obtained additional insights into the mechanism underlying the inhibition of YcgO by unphospho-PtsN. We show that PtsN displays a phosphorylation state–dependent interaction with YcgO. Unphospho-PtsN is capable of physically interacting with YcgO, which is markedly attenuated upon phosphorylation of PtsN. A cytoplasmic C-terminal region (CTR) of YcgO is identified and of the two putative subdomains in the CTR; the carboxy-terminal CorC domain is identified as the site of interaction, with unphospho-PtsN. A second subdomain in the CTR, regulator of conduction of K^+^ (RCK), may function as an activator of YcgO-mediated K^+^ efflux. Our studies indicate that the unphospho–PtsN:CorC interaction may negatively impact the RCK-mediated activation of YcgO, leading to the inhibition of YcgO. Lastly, we show that YcgO functions as a K^+^/H^+^ antiporter in everted vesicles.

## Results

### A limited *in vivo* membrane topology analyses identifies a two-domain architecture of YcgO

**Figure 1:**
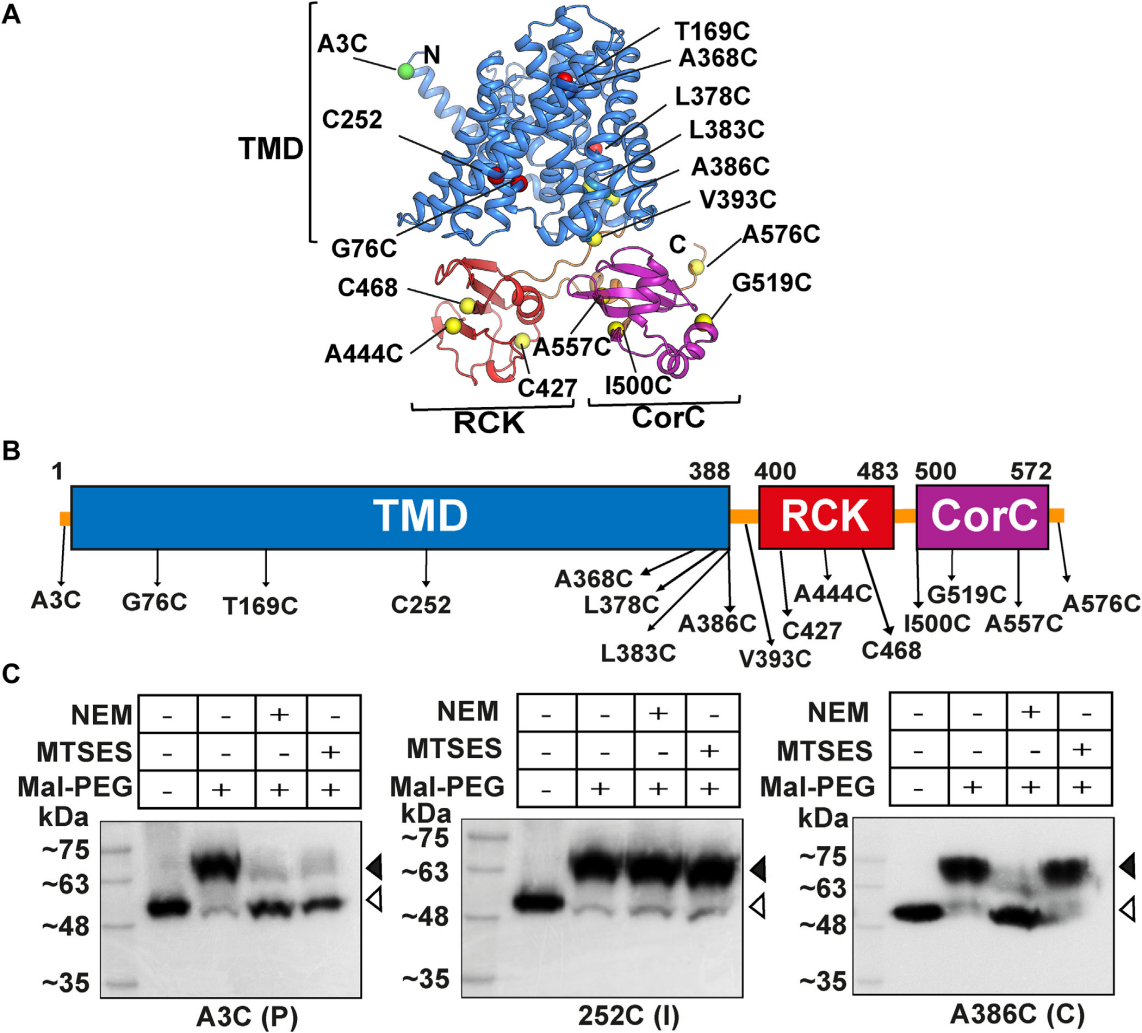
**The AlphaFold2 predicted structure of YcgO, distribution of cysteine (Cys) residues planted in Cysless YcgO, and the accessibility of certain Cys residues.***A*, PyMOL rendition of the AlphaFold2 model of YcgO. Amino acid segments corresponding to the predicted transmembrane domain (TMD; 1–388), the RCK domain (400–483), and the CorC (500–572) domain are colored in *blue*, *light red*, and *purple*, respectively. The N and C-termini are marked. The various Cys substitutions placed in Cysless YcgO are colored according to their reactivity to NEM alone (*yellow spheres*) and NEM and MTSES (*green sphere*). NEM/MTSES nonreactive cysteines are indicated as *red spheres*. *B*, schematic depicting the distribution of Cys [C] substitutions in Cysless YcgO. Cysteines at amino acid number 252, 427, and 468 are the natural cysteines in YcgO. The three domains of YcgO colored *blue*, *red*, and *purple*, are indicated (to scale). The 3× FLAG and 6× His epitopes abutted to the C-terminus of Cysless YcgO are not indicated. *C*, anti-FLAG immunoblots showing modification of the indicated mono-Cys derivatives of the Cysless YcgO following premodification with NEM and MTSES. Mid-exponential phase cultures of the strain GJ18836 bearing plasmids (Table S2) expressing the A3C, 252C, and A386C derivatives of Cysless YcgO were processed as described in the SI methods section. Treatments with NEM, MTSES, and Mal-PEG are indicated and the positions of free (*white triangles*) and Mal-PEG adducts (*black triangles*) are marked. The topological location of the Cys residues is indicated in parenthesis. P, I, and C denote periplasmic, intramembrane, and cytoplasmic, respectively.

Analysis of the amino acid sequence of YcgO on InterPro (25) revealed the existence of three distinct domains in YcgO, namely (i) a cation/H^+^ antiporter domain spanning amino acid residues 16 to 388, (ii) an RCK_C (RCK) domain contributed by the 403 to 485 segment, and (iii) a CorC_HlyC (CorC) domain linked to the RCK domain through a linker in the 492 to 573 segment. To obtain insights into the *in vivo* membrane organization of YcgO, we sourced the AlphaFold2 (AF2) prediction (26) of YcgO (UniProt ID P76007, Fig. 1*A*). Consistent with the output from InterPro, the AF2 model predicts that YcgO may comprise a transmembrane domain (TMD) containing the N-terminal 388 amino acids and a CTR of 190 amino acids. The TMD bears 13 α-helical transmembrane segments (TMSs). Two putative β-sheet rich subdomains, resembling the RCK and the CorC type transporter-associated domain, in the CTR are discerned. The RCK and the CorC domains span respectively the 400 to 483 and the 500 to 572 amino acid regions in the CTR. The two domains are connected by a short 15 amino acid α-helical linker. Regulator of conduction of K^+^ domains constitute the components of both prokaryotic and eukaryotic K^+^ transporters/channels and regulate transmembrane K^+^ flux (27, 28), whereas the CorC domain is located at the C-termini of some prokaryotic magnesium (Mg^2+^) transporters (29, 30, 31). The TMD of YcgO resembles a typical CPA1 fold, with a probable periplasmic N-terminus and a cytoplasmic C-terminus. The K^+^/H^+^ recognition is likely to be localized to the acidic residues in the vestibule, namely D133, E155, E157, and D162, and is similar to the architecture of some well-characterized Na^+^/H^+^ antiporters like MjNhaP and PaNhaP (32, 33). The RCK domain is predicted to possess a fold similar to the structurally elucidated RCK domain of the TrkA octameric gating ring of the *Thermotoga maritima* Trk K^+^ transporter (PDB ID: 3JXO, (34)), while the CorC domain is predicted to resemble the corresponding domains in structurally elucidated putative Mg^2+^ and Co^2+^ efflux proteins (PDB IDs: 2PLI, 3LLB and 4HG0).

Towards corroboration of the AF2 model of YcgO, we performed cysteine (Cys) accessibility studies (35, 36) to assess the *in vivo* membrane topology of YcgO. We constructed a derivative of *ycgO*, encoding the protein, YcgO_FH_, bearing a C-terminal bipartite 3× FLAG hexahistidine tag, expressed from the IPTG inducible P_*trc*_ promoter on the plasmid pHYD5011. The C252A, C427A, and C468A substitutions were introduced in YcgO_FH_, to generate a cysteine (Cys)-less derivative of YcgO_FH_, YcgO_CL_. Nine mono-Cys substitutions in the putative CTR and seven in the putative TMD were introduced into YcgO_CL_ (Fig. 1, *A* and *B*). Expression of *ycgO* leads to growth inhibition of the Δ*ptsN* mutant in media with high (115 mM, K_115_), but not low (1 mM, K_1_) [K^+^]_e_s (15). This growth inhibition correlates with K^+^ limitation mediated by YcgO in the absence of unphospho-PtsN (15, 23). The activity of the inducible high affinity K^+^ uptake system Kdp is thought to mitigate the K^+^ limitation in K_1_ medium. The aforementioned K^+^-limited growth phenotype of the Δ*ptsN* mutant is referred to as K^L^, standing for K^+^ limited growth.

Basal level expression of YcgO_FH_, YcgO_CL_, and all its mono-Cys substitution derivatives from the P_*trc*_ promoter of the plasmid pTrc99A led to the K^L^ in the Δ*ptsN* Δ*ycgO* double mutant JD509 (Fig. S1). JD509 bearing pTrc99A did not display the K^L^; it grew equally well both on K_1_ and K_115_ media (Fig. S1). This indicated that all of the employed derivatives of YcgO_CL_ were functional *in vivo*.

To infer the topological location of a given Cys residue in YcgO_CL_, cells of the strain GJ18836 that lacks endogenous YcgO, expressing the various mono-Cys–substituted YcgO_CL_ derivatives, were pretreated separately with NEM and MTSES. The inner membrane permeable NEM reacts with Cys residues with periplasmic and cytoplasmic exposure, whereas the impermeable MTSES reacts only with Cys residues exposed to the periplasm. Cys residues facing the hydrophobic core of the membrane cannot react with NEM and MTSES. Any Cys residue that undergoes modification either by NEM or MTSES cannot react with Mal-PEG in the presence of SDS. Mal-PEG adduct formation with a free Cys leads to increase in the molecular weight of the protein by approximately 5 kDa. The free and the Mal-PEG bound species of a protein can be detected with immunoblotting with anti-FLAG antibody following SDS PAGE of samples. In these studies, we noted the mono-Cys derivatives of YcgO_CL_ displayed anomalous migration in SDS PAGE gels, migrating as an approximately 54 kDa species despite its theoretical molecular weight being 65.7 kDa (Fig. 1*C*). This is also seen in all immunoblots pertaining to detection of 3× FLAG tagged YcgO. Representative images of immunoblots of three mono-Cys substituted derivatives of YcgO_CL_ bearing Cys residues at the third, 252nd and 386th amino acids subjected to the aforementioned Cys modification regimen are shown in Figure 1*C*. These images permitted the inference that the third, 252nd and 38 amino acids of YcgO display respectively periplasmic, membrane-embedded and cytoplasmic location. A C-terminal cytoplasmic region (CTR) of roughly 195 amino acids in length could be discerned, extending from amino acid 384 to 578, as all interrogated Cys substitutions in this stretch were prone to modification with NEM but not with MTSES (Fig. S2). The partial reactivity of Cys 383 with NEM indicates that this residue most likely resides at the cytoplasm/inner membrane interface (Fig. S2). Preceding the CTR is the last (13th as per the AF2 model) TMS, supported by the observation that Cys 368 and Cys 378 display an intramembrane location (Fig. S2). We could also assign an intramembrane location to cysteines placed at the 76th and the 169th amino acid position in YcgO (Fig. S2).

Overall, these topological analyses show that the membrane organization of YcgO coheres well with AF2 model and is consistent with a two-domain architecture, that comprises an approximately 383 amino acid long N-terminal TMD linked to a 195 amino acid long cytoplasmic CTR. The overall topology of YcgO is indicative of an N_OUT_-C_IN_ configuration.

In these studies we observed for multiple Cys substituted proteins variations in the band intensities of YcgO:Mal-PEG adducts (see Fig. S2, G76C, 427C and A368C for examples). Furthermore, variations were also apparent for some Cys substitutions in duplicate experiments. While these may arise due to inefficient transfer to the blotting membrane of these adducts, it does not interfere with assigning a location to a given Cys residue. In certain instances for example Cys 169 (Fig. S2), despite the appearance of a YcgO:Mal-PEG adduct some amount of free YcgO species was detected which may be attributed to reduced reactivity of Cys 169. Lastly, far-UV circular dichroism (CD) of a purified carboxy-terminal hexahistidine tagged CTR (CTR_His_) spanning amino acids 386 to 578, revealed that significant portion of CTR_His_ existed as β-sheet, with moderate and low random coil and α-helical content respectively (Fig. S3), which is consistent with the predicted secondary structure features of the CTR in the AF2 model.

### Constitutive YcgO activity elicited by amino acid substitutions in the CTR and the TMD of YcgO and by the removal of the CorC domain

**Figure 2:**
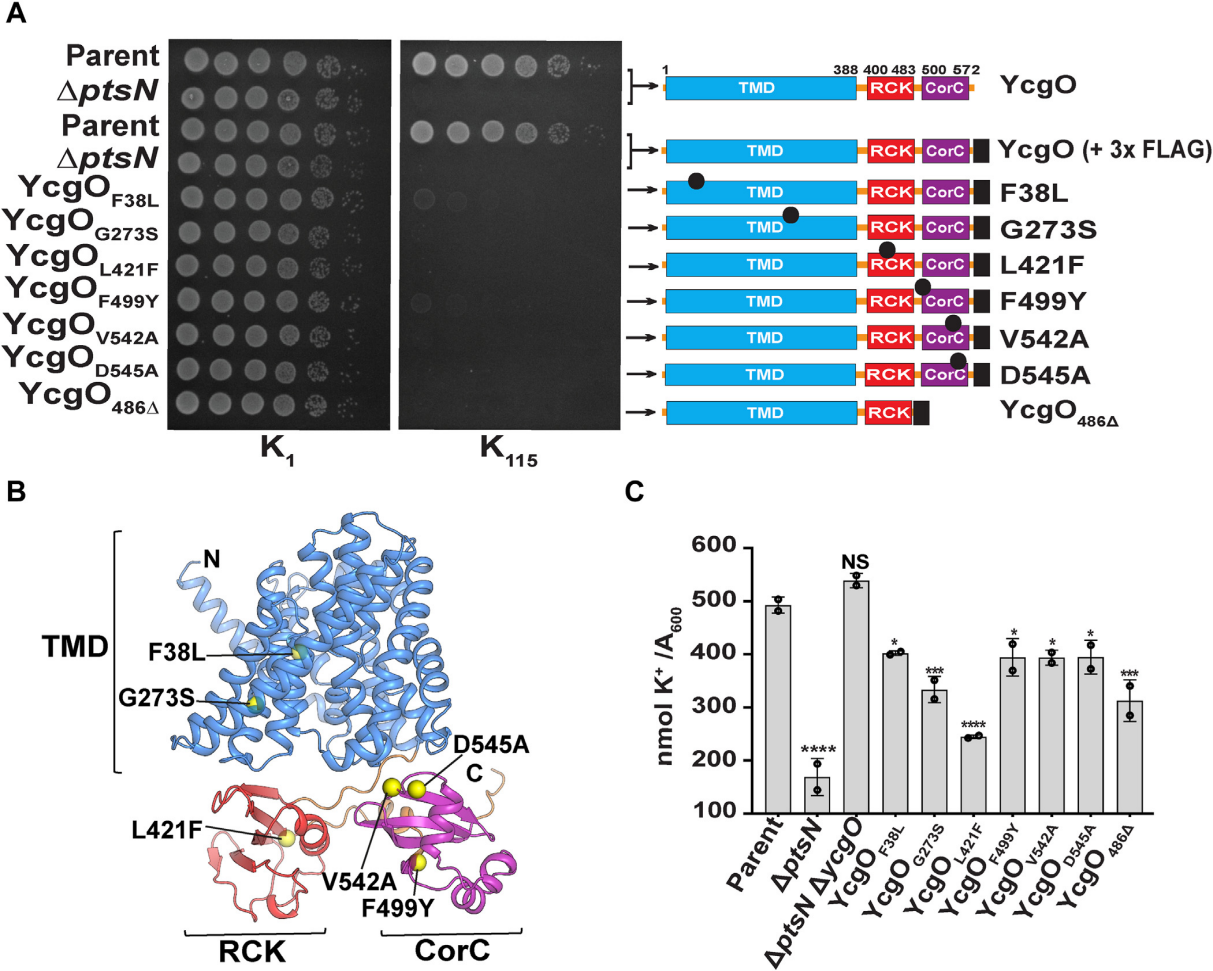
**Growth phenotypes of amino acid substitutions in YcgO that lead to the YcgO**^**Con**^**phenotype, their distribution in YcgO, the YcgO**^**Con**^**phenotype elicited by YcgO**_**486Δ**_**, and cellular K**^**+**^**content in strains expressing YcgO**^**Con**^**proteins.***A*, ten-fold serial dilutions of the parent and its Δ*ptsN* derivative expressing WT and 3× FLAG tagged YcgO (YcgO) and the parent expressing the indicated C-terminally 3× FLAG tagged YcgO bearing the YcgO^Con^ substitutions or the CorC deletion bearing proteins were spotted on the surface of minimal glucose agar plates of [K^+^]_e_ 1 mM (K_1_ agar) and 115 mM (K_115_ agar). The domain organization (to scale) in each YcgO-expressed protein is indicated adjacent to the spotting. *Black filled circles* indicate the position of the YcgO^Con^ substitution and the *black box* represents the 3× FLAG epitope, drawn not to scale. *B*, distribution of the YcgO^Con^ amino acid substitutions (*yellow spheres*) on the AlphaFold2 model of YcgO. Domain coloring is as per Figure 1*A*. *C*, K^+^ content/*A*_*600*_ of the parent, its Δ*ptsN*, Δ*ptsN* Δ*ycgO*::*cat* (Δ*ycgO*) derivatives, and the parent expressing the indicated YcgO^Con^ proteins, following transient exposure to K_115_ medium. The expressed YcgO protein in all strains bears a C-terminal 3× FLAG tag. Results represent mean ± SD of measurements conducted in duplicates on two independent cultures. Statistical significance was determined using ordinary one-way ANOVA (multiple comparisons test). The *p*-value symbol “∗∗∗∗,” “∗∗∗,” and “∗” represent, respectively *p* < 0.0001, *p* ≤ 0.0002, and *p* ≤ 0.0332 and NS (*p* > 0.1234) indicates a nonsignificant change. Strains used in panels A are GJ17829, GJ17831, GJ17854, GJ19263, GJ19264, GJ19265, GJ19266, GJ19267, GJ19290, GJ19291, and GJ22207 and those in *panel C* are without GJ17829, GJ17831 but contain in addition GJ22206.

Under the scenario that unphospho-PtsN negatively regulates YcgO activity, one should be able to identify a class of YcgO mutant proteins whose expression phenocopies the Δ*ptsN* mutant. That is these proteins would yield the K^L^ even in a *ptsN*^+^ strain, being rendered immune to the unphospho-PtsN imposed block and would be constitutively active, yielding the YcgO^Con^ phenotype. To isolate missense mutations in *ycgO* that yield the YcgO^Con^ phenotype, we used an approach that involved performing error prone PCR on the *ycgO* gene present on a single copy plasmid pMU575 (37). Following a genetic screening procedure, we employed steps of site directed mutagenesis, to establish association of a given *ycgO* missense mutation with the YcgO^Con^ phenotype. Furthermore, the missense mutations leading to the Ycgo^Con^ phenotype were recombined into the chromosome of a suitable strain. In the chromosomal configuration both the wild type YcgO and YcgO^Con^ proteins were appended with a 3× FLAG tag to their C-termini. Chromosomal expression of all YcgO^Con^ proteins led to the YcgO^Con^ phenotype (Fig. 2*A*). Expression levels of the YcgO^Con^ proteins were comparable to the wild-type YcgO (Fig. S4*A*). Detailed procedures pertaining to the above are described in supporting information (SI) methods. In this study the C-terminal 3× FLAG tagged YcgO, expressed from the chromosome and retaining YcgO function ((15), Fig. 2*A*) is referred to as YcgO and its YcgO^Con^ substitution derivatives are indicated with a subscripted amino acid substitution numbering.

We identified two amino acid substitutions in the TMD, namely F38L and G273S, and four amino acid substitutions in the CTR whose expression in the *ptsN*^+^ strain GJ17829, led to the YcgO^Con^ phenotype, that is GJ17829 expressing the YcgO^Con^ proteins displayed the K^L^, a growth phenotype resembling the Δ*ptsN* mutant (Fig. 2, *A* and *B*). Of the four substitutions in the CTR, two lie in the CorC domain, namely V542A and D545A. The F499Y substitution is at the N-terminal edge of the CorC domain whereas L421F lies in the RCK domain (Fig. 2, *A* and *B*). The YcgO^Con^ phenotype of these proteins correlated with K^+^ limitation in K_115_ medium, as overexpression of the K^+^ uptake protein Kup, alleviated the inability of the Δ*ptsN* mutant and strains expressing YcgO^Con^ proteins to grow in K_115_ medium (Fig. S5). Lastly, we measured cellular K^+^ content in the strain GJ17854 that expresses 3× FLAG tagged YcgO, its Δ*ptsN*, Δ*ptsN* Δ*ycgO* (Δ*ycgO*;;*cat*) derivatives and in derivatives of the parent GJ17829, expressing 3× FLAG tagged YcgO^Con^ proteins, after a transient (3 h) exposure to K_115_ medium, following their growth to mid-exponential phase in K_1_ medium. In comparison to the GJ17854, the Δ*ptsN* mutant displayed reduced cellular K^+^ content (Fig. 2*C*). Furthermore, all YcgO^Con^ mutation bearing strains had cellular K^+^ contents that were lower than the parent, but to varying extents (Fig. 2*C*). As noted earlier (15), absence of YcgO, in the Δ*ptsN* mutant, led to elevation in the cellular K^+^ content, to a level equivalent to the parent (Fig. 2*C*).

The YcgO^Con^ phenotype was also elicited by chromosomal expression of a derivative of YcgO, YcgO_486Δ_, lacking the terminal 92 amino acids of YcgO, that removes the CorC domain (Fig. 2*A*), that was associated with reduced cellular K^+^ content (Fig. 2*C*) and was alleviated by expression of Kup (Fig. S5). YcgO_486Δ_ was expressed at slightly lower levels in comparison to YcgO (Fig. S4*B*).

During the course of studies on the YcgO^Con^ mutations, we noted that expression of all YcgO^Con^ proteins yielded the YcgO^Con^ phenotype only in a genetic background wherein the Trk K^+^ uptake transporter was disabled by the removal of the TrkA subunit (38). This was not the case with the Δ*ptsN* mutant (Figs. S6 and S7). This aspect of the YcgO^Con^ proteins is alluded to in the discussion section.

### The L421S and P438Q substitutions in the RCK domain impair YcgO function

**Figure 3:**
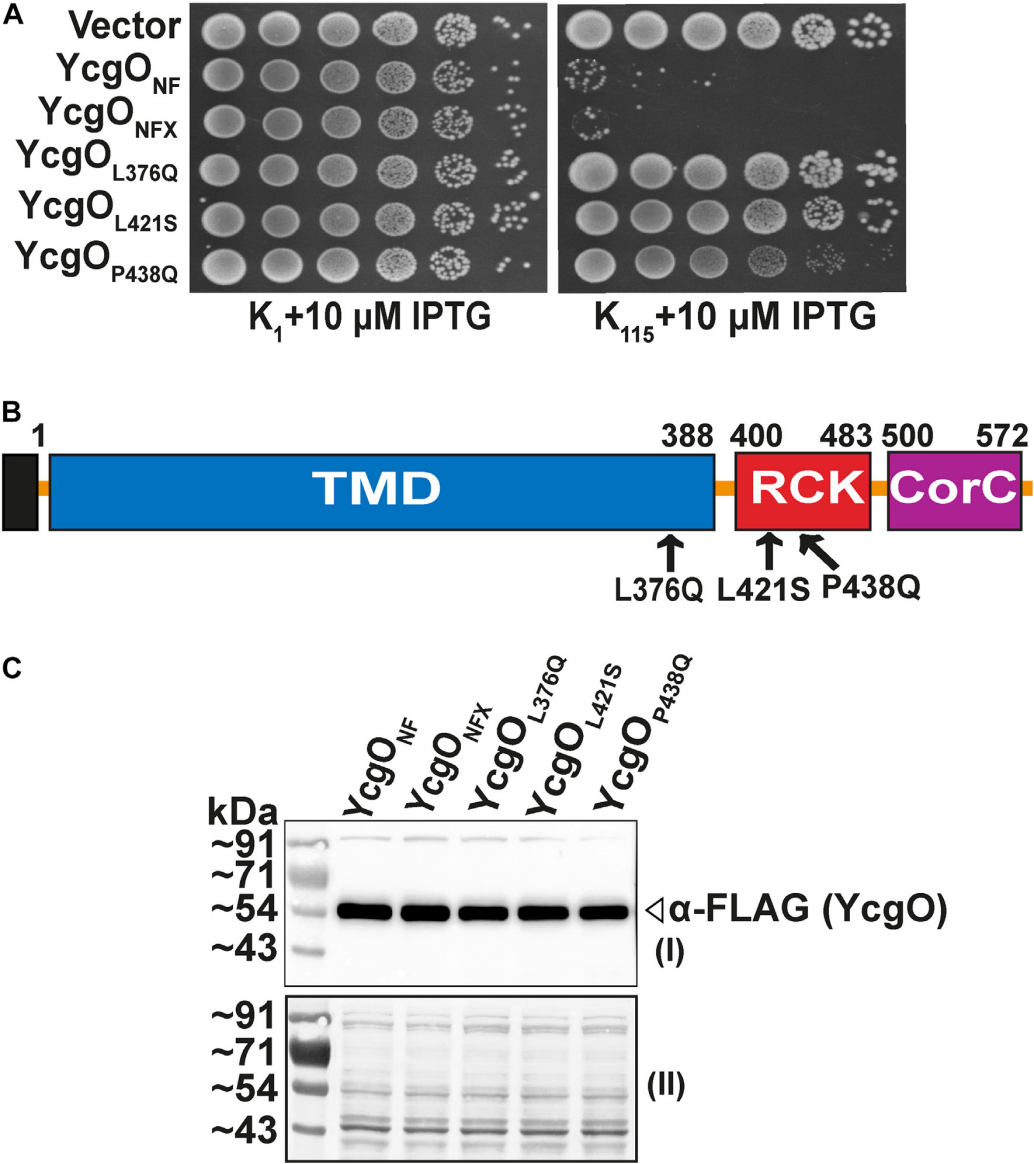
**Growth phenotypes and expression level of the indicated YcgO impairing amino acid substitutions in the CTR.***A*, serial dilutions of cultures of the Δ*ptsN* Δ*ycgO*::*cat* double mutant GJ22206 bearing the plasmid pTrc99A (vector) and its derivative plasmids expressing YcgO_NF_, YcgO_NFX_ and plasmids expressing YcgO_NFX_ with the indicated amino acid substitutions were plated on K_1_ and K_115_ agar plates containing 10 μM IPTG. *B*, schematic depicting the distribution of YcgO disabling amino acid substitutions in or near the RCK domain. Protein domains of the YcgO_NFX_ (and YcgO_NF_) polypeptides are indicated (to scale), and the *black* box (not to scale) represents the N-terminally abutted 3× FLAG tag. *C*, anti-FLAG immunoblots, depicting expression levels of the aforementioned proteins (*white triangle*). Cell extracts prepared from *A*_*600*_ normalized cultures were electrophoresed on SDS-PAGE, transferred to a PVDF membrane, and probed with anti-FLAG antibody (I). A portion of the membrane stained with amido-black (II) as a loading control is indicated. Plasmids employed are listed in Table S2.

We constructed a derivative of *ycgO* that encoded a protein, YcgO_NF_ bearing an N-terminally abutted 3× FLAG tag. In this *ycgO* variant, a synonymous substitution of the 358th codon was generated, which created a restriction site for mutagenesis purposes. The encoded protein is designated YcgO_NFX_. Growth tests showed both YcgO_NF_ and YcgO_NFX_ retained YcgO function (Fig. 3*A*). Introduction of the aforementioned restriction site, permitted selective mutagenesis of a region of *ycgO*, spanning the 358th to 578th codons that encodes the entire CTR plus a portion of the terminal (13th as per the AF2 model) TMS. Details on the procedures for performing the aforementioned mutagenesis, and the procedure for recovery of YcgO impairing amino acid substitutions, are described in SI methods. We identified three amino acid substitutions, one located in the terminal TMS (L376Q), and two (L421S, P438Q) in the RCK domain in YcgO_NFX_ that alleviated the K^L^ imparted by YcgO_NFX_ in the Δ*ptsN* Δ*ycgO* double mutant GJ22206 (Fig. 3, *A* and *B*). The P438Q substitution led to partial loss of YcgO function (Fig. 3*A*). Immunoblotting with anti-FLAG antibody showed that the aforementioned substitution bearing proteins were expressed at levels comparable to YcgO_NFX_ (Fig. 3*C*). These observations indicate that besides the CorC domain in CTR, that negatively regulates YcgO, the CTR may possess a TMD activating capacity conferred by the RCK domain. The L376Q substitution may exert an indirect effect to perturb RCK function.

### YcgO displays phosphorylation state dependent interaction with PtsN

**Figure 4:**
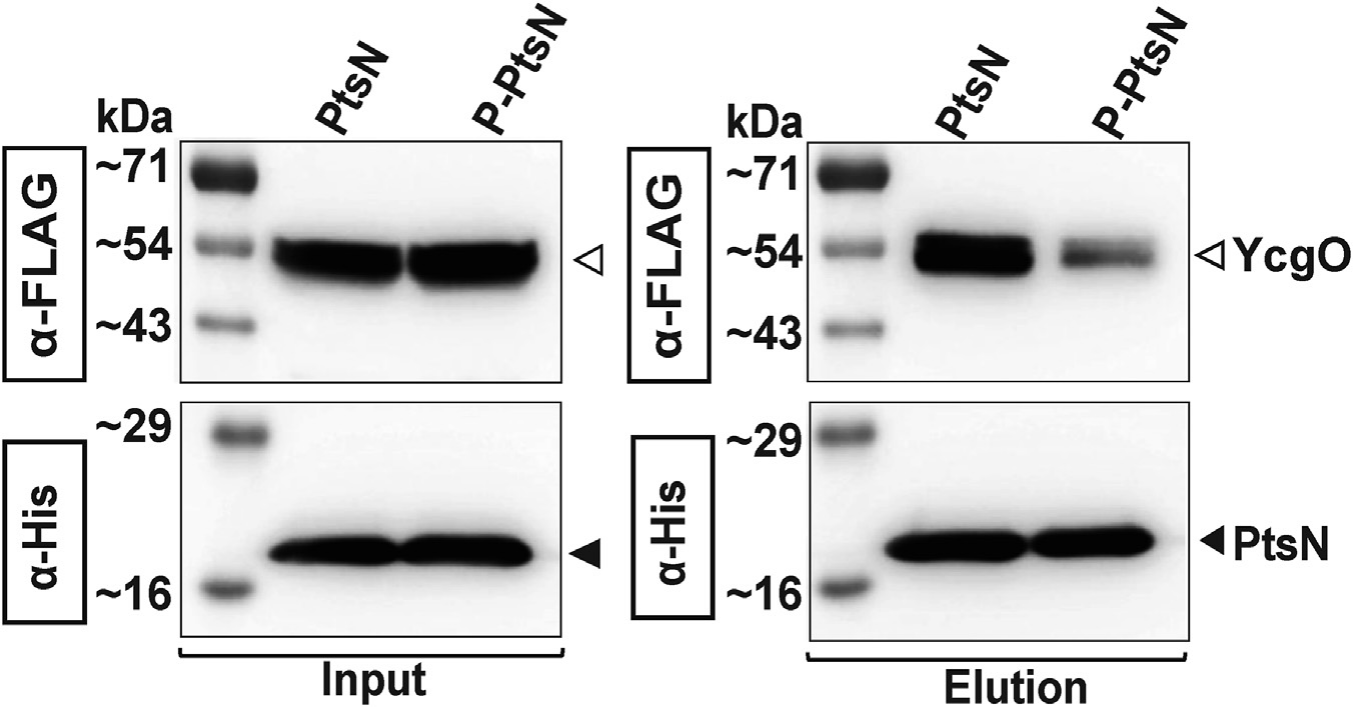
**PtsN displays phosphorylation state–dependent interaction with YcgO.** Immunoblots depicting the copurification propensity of 3× FLAG-tagged YcgO (YcgO) with preparations of unphospho-PtsN_His_ (PtsN) and phospho-PtsN_His_ (P-PtsN). Copurifications were performed as described in the experimental procedures section. *A*_*600*_ normalized volumes of DDM-solubilized cell extracts of the strain GJ21020 expressing YcgO, following exposure to PtsN and P-PtsN (Input) and appropriate volumes of the Ni-NTA eluates, adjusted for the western intensity of the PtsN_His_ species (Elution) were subjected to SDS-PAGE and immunoblotting as described in the experimental procedures and SI methods. Anti-FLAG and anti-His immunoblots for the detection of YcgO and PtsN proteins respectively are marked with rectangular boxes. *White* and *black triangles* mark the YcgO and the PtsN species, respectively.

To test whether unphospho-PtsN can interact with YcgO, we gauged the propensity of YcgO to co-purify, with unphospho-PtsN. For this purpose, a C-terminally hexahistidine tagged PtsN (PtsN_His_), was expressed from the P_*trc*_ promoter of the plasmid pHYD5006 (15) following induction of the P_*trc*_ promoter with 1 mM IPTG, in the strain GJ21015 that lacks PtsP and PtsN. In the Δ*ptsP* mutant PtsN exists solely in the unphospho-state (39, 40). The overproduced PtsN_His_ was then purified by Ni-NTA affinity chromatography. LC ESI-MS analysis of PtsN_His_ purified from the Δ*ptsP* strain, revealed the absence of phosphorylated species (SI methods, Fig. S8*A*). Purified unphospho-PtsN_His_ was mixed with DDM solubilized cell extracts of GJ21019 and GJ21020. The former and the latter strains express respectively chromosomally encoded versions of two inner membrane proteins appended with C-terminal 3× FLAG tags, namely the disulfide oxidoreductase DsbB (41) and YcgO. Following purification of unphospho-PtsN_His_, the input fraction and Ni-NTA resin eluates were subjected to SDS PAGE followed by immunoblotting with anti-FLAG and anti-His antibodies. We found that YcgO but not DsbB copurified with unphospho-PtsN_His_ (Fig. S9). Furthermore, co-purification of YcgO was detected only when unphospho-PtsN_His_ was added to the lysate (Fig. S9). YcgO did not co-purify with another C-terminally hexahistidine tagged cytoplasmic protein, NusG_His_ ((42), Fig. S10). To assess the propensity of the phosphorylated version of PtsN, phospho-PtsN, to interact with YcgO, we devised a procedure for purification of PtsN_His_ that would be enriched with phospho-PtsN_His,_ described in SI methods. During this purification procedure we were able to separate and purify phospho-PtsN_His_ and unphospho-PtsN_His_ (Fig. S11). In co-purification procedures we detected a marked reduction in the propensity of YcgO to co-purify with a PtsN_His_ preparation that largely contains the phosphorylated species (Fig. 4). Co-purification with this species may occur either due to dephosphorylation of phospho-PtsN_His_ by cellular phosphatases in the cell lysate or due to the presence of contaminating amounts of unphospho-PtsN in the preparation of phospho-PtsN.

### Absence of the CorC domain leads to loss of interaction with unphospho-PtsN

**Figure 5:**
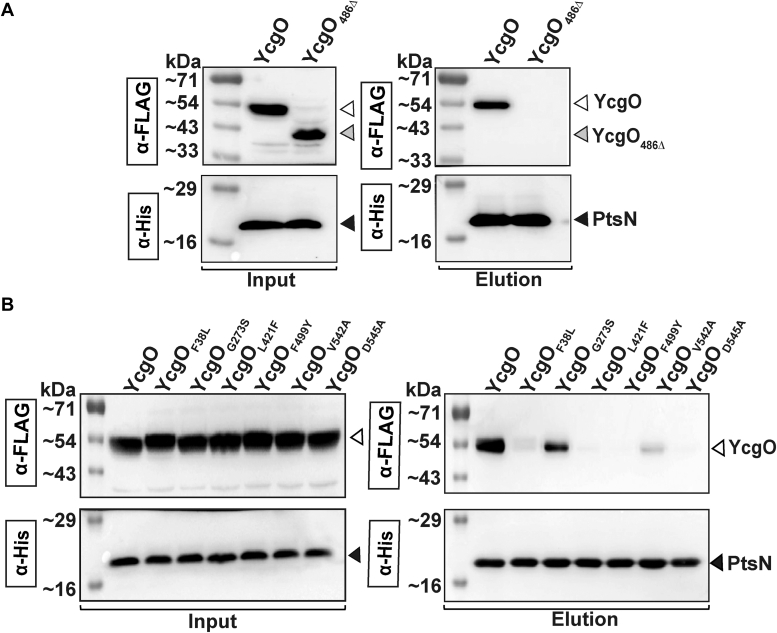
**Absence of the interaction of unphospho-PtsN with YcgO lacking the CorC domain and with derivatives of YcgO bearing the indicated YcgO**^**Con**^**substitutions in the CTR.** Copurification procedures was applied to gauge the propensities of the indicated 3× FLAG-tagged YcgO proteins to interact with unphospho-PtsN_His_ (PtsN) in panels A and B. All sample processing procedures are as per Figure 4. Anti-FLAG and anti-His immunoblots for the detection of YcgO and PtsN proteins are marked with rectangular boxes. In panel A for YcgO and YcgO_486Δ_, cell extracts were prepared from appropriate cultures with an *A*_*600*_ of 50 and 75, respectively. Cell extracts of the following strains were used: GJ22204, GJ21020 (for (*A*)) and GJ21020, GJ21021, GJ21022, GJ21023, GJ21024, GJ22211, and GJ22212 (for (*B*)). In *panel A*, *white* and *light gray triangles* mark the two YcgO proteins and the *black triangle* marks unphospho-PtsN_His_. In *panel B*, YcgO and its derivatives in the immunoblot images are marked with a *white triangle* and the *black triangle* marks unphospho-PtsN_His_.

We tested the propensity of YcgO_486Δ_ to interact with unphospho-PtsN_His_ and noted that YcgO_486Δ_ did not co-purify with unphospho-PtsN_His_ (Fig. 5*A*). Additional experimentation revealed that four YcgO^Con^ proteins bearing YcgO^Con^ substitutions in the CTR, either did not interact with unphospho-PtsN_His_ (Fig. 5*B*), or formed a very weakened complex, for example YcgO_V542A_ (Fig. 5*B*). Of the two YcgO^Con^ substitutions in the TMD YcgO_G273S_ but not YcgO_F38L_ displayed an interaction with unphospho-PtsN_His_ (Fig. 5*B*).

### Detection of unphospho-PtsN:YcgO complex *in vitro*

**Figure 6:**
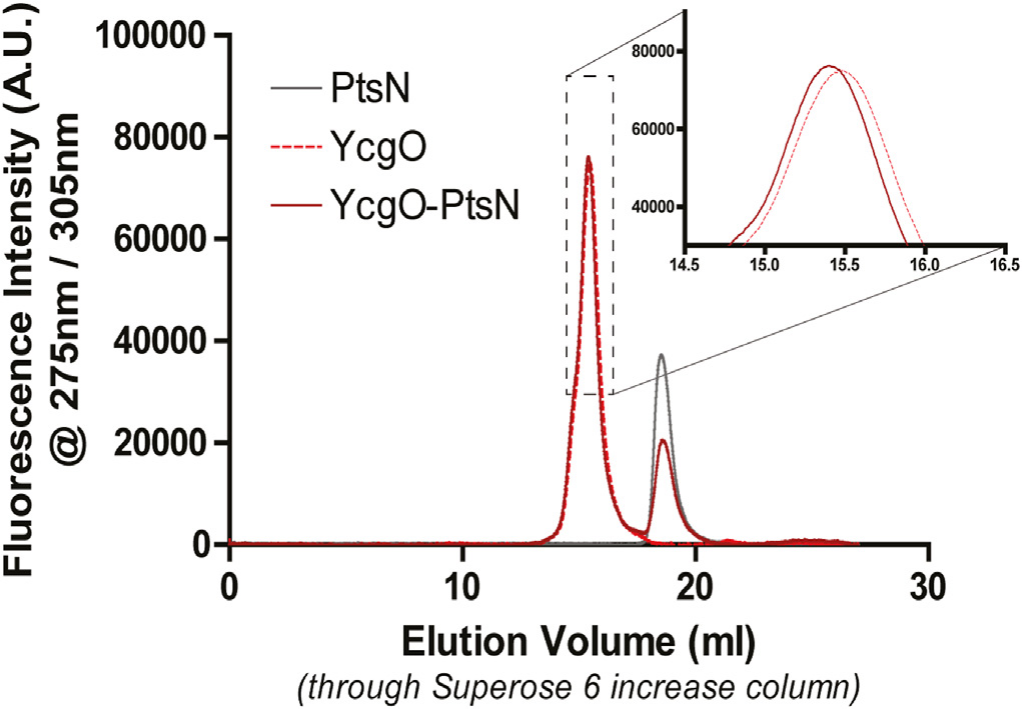
**Unphospho-PtsN:YcgO complex formation *in vitro*.** Fluorescence detection size-exclusion chromatography profile of YcgO_FH_ (YcgO) run apo or in complex with unphospho-PtsN_His_ (PtsN), *dotted* and *solid red* traces, respectively. Profile of the apo PtsN run is depicted in *gray* trace. Inset depicts an enlarged view of YcgO elution peak to emphasize the leftward shift of the same when run with PtsN in molar excess in the mix, suggesting complex formation. Data shown for YcgO_FH_: unphospho-PtsN_His_ in 1:5 M ratio for one trial of two biological replicates.

Unphospho-PtsN_His_;YcgO_FH_ interaction *in vitro* was assayed with purified protein samples through FSEC (fluorescence detection size exclusion chromatography, SI methods) in a buffer with 100 mM KCl and 100 mM NaCl. Tyrosine fluorescence (λ_ex/_λ_em_ = 275 nm/305 nm) was used to detect both proteins as PtsN_His_ lacks tryptophan residues. The leftward shift of the tyrosine peaks at ∼15 to 15.5 ml range, which corresponds to the elution volume of YcgO_FH_, is indicative of the formation of the unphospho-PtsN_His_:YcgO_FH_ complex (inset, Fig. 6). The smaller peak at 18.5 ml in tyrosine fluorescence trace corresponds to unphospho-PtsN_His_ (Fig. 6). Co-purification of YcgO in cell extracts with unphospho-PtsN was observed in a buffer containing 100 mM NaCl and 100 mM KCl (Fig. S12).

### A K^+^/H^+^ antiport activity is elicited in everted vesicles bearing overexpressed YcgO

YcgO is a member protein of the CPA1 family monovalent cation/proton exchangers (21, 43). Orthologs of YcgO, Vp-NhaP2 and Vc-NhaP2 from *Vibrio parahaemolyticus* and *Vibrio cholerae* respectively have been shown to mediate K^+^/H^+^ antiport (44, 45). We tested whether YcgO mediated K^+^/H^+^ antiport activity can be detected in everted membrane vesicles. For this purpose, the plasmid pHYD6578 encoding C-terminally 3× FLAG tagged YcgO, expressed form the L-arabinose (Ara) inducible P_*ara*_ promoter, and the vector, pBAD24 (46), were introduced into the strain GJ22224. GJ22224 lacks KefB, KefC, ChaA, YbaL, YcgO, PtsN and NhaA. ChaA and NhaA mediate respectively K^+^/H^+^ and Na^+^/H^+^ antiport (47, 48) whereas YbaL is considered to be a paralog of the KefB and the KefC K^+^/H^+^ antiporters. We noted that joint removal of NhaB a paralog of NhaA (49), and NhaA in GJ22224 led to growth sickness, hence studies have been performed with GJ22224 that contains an intact copy of *nhaB*. Everted vesicles were prepared from GJ22224 bearing pHYD6578 and pBAD24 (vector) following their cultivation in the presence of 0.05% Ara. We tested for K^+^ and Na^+^ mediated proton release in everted vesicles in aliquots that were pre-exposed to the pH sensitive dye ACMA and their lumens pre-loaded with protons following treatment with ATP. Proton entry into the lumen is facilitated by the activity of F_0_F_1_ ATPase. Quenching of ACMA fluorescence was taken as an indicator of establishment of the proton gradient.

**Figure 7:**
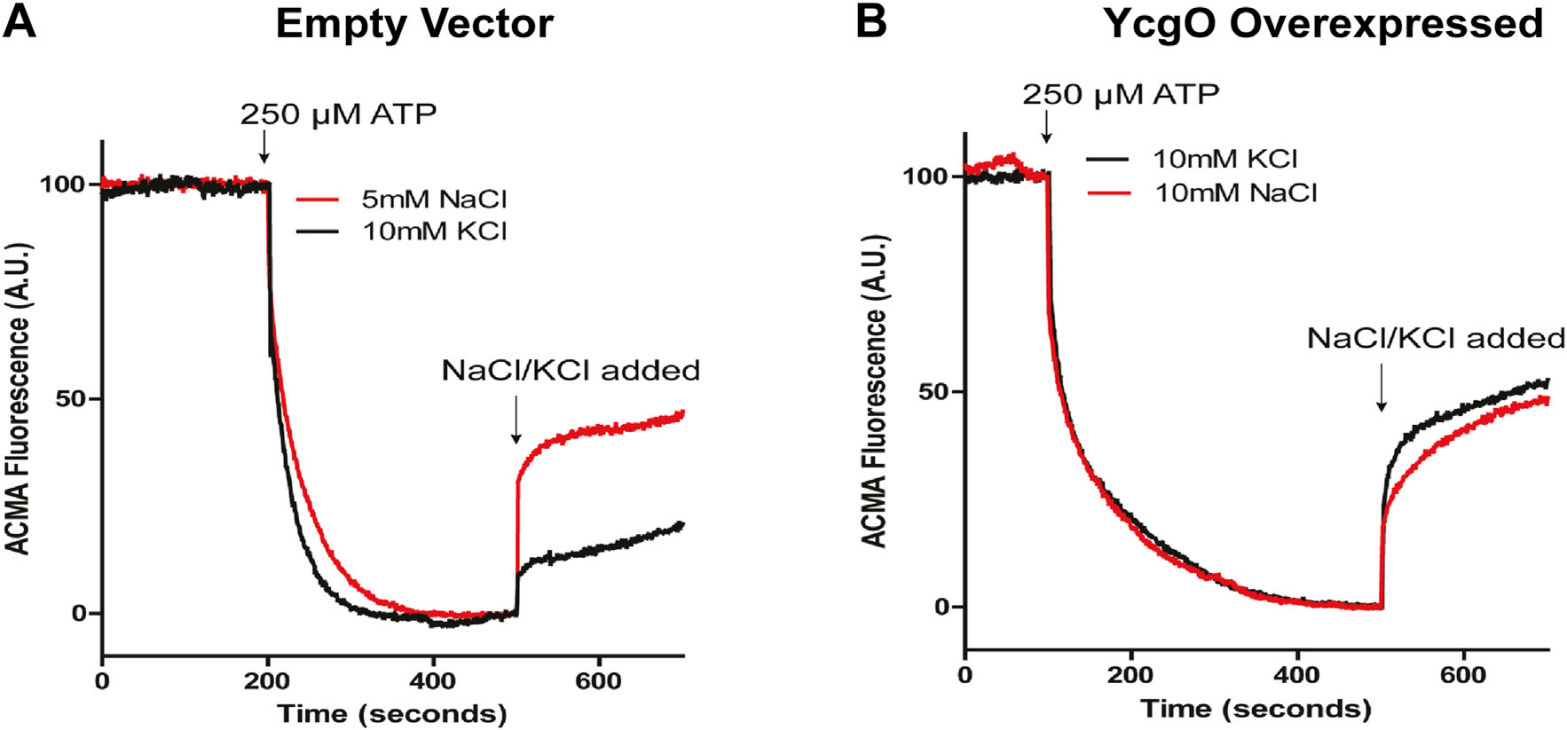
**K**^**+**^**/H**^**+**^**antiport activity of YcgO.** Everted vesicle-based transport assay run with vesicles derived from the strain GJ22224 bearing the plasmids pBAD24 (Empty vector) (*A*) and the plasmid pHYD6578 (YcgO overexpressed) (*B*). Traces correspond to pH-dependent ACMA fluorescence measured at λ_ex/em_ = 409 nm/474 nm. *Red* and *black traces* correspond to assays performed with either NaCl or KCl at the indicated concentrations, as substrates respectively. One trace is shown for each trial; assays were performed with two independent batches of vesicles and ≥3 technical replicates.

In vesicles derived from GJ22224, bearing the vector following the establishment of the proton gradient, addition of 5 mM NaCl provoked proton extrusion as gauged by the dequenching of ACMA fluorescence, whereas addition of 10 mM KCl led to modest dequenching of ACMA fluorescence (Fig. 7*A*). In vesicles prepared from the GJ22224 bearing pHYD6578, addition of 10 mM KCl provoked a dequenching of ACMA fluorescence of a much larger magnitude in comparison to that observed for vesicles derived from GJ22224 bearing the vector. Sodium at 10 mM also provoked proton efflux in everted vesicles bearing overexpressed YcgO (Fig. 7*B*). Sodium ion provoked proton efflux can be attributed to the presence of NhaB activity, in this strain, whereas increased K^+^ ion promoted proton flux under the condition of YcgO overexpression is supportive of a K^+^/H^+^ antiporter function of YcgO.

## Discussion

**Figure 8:**
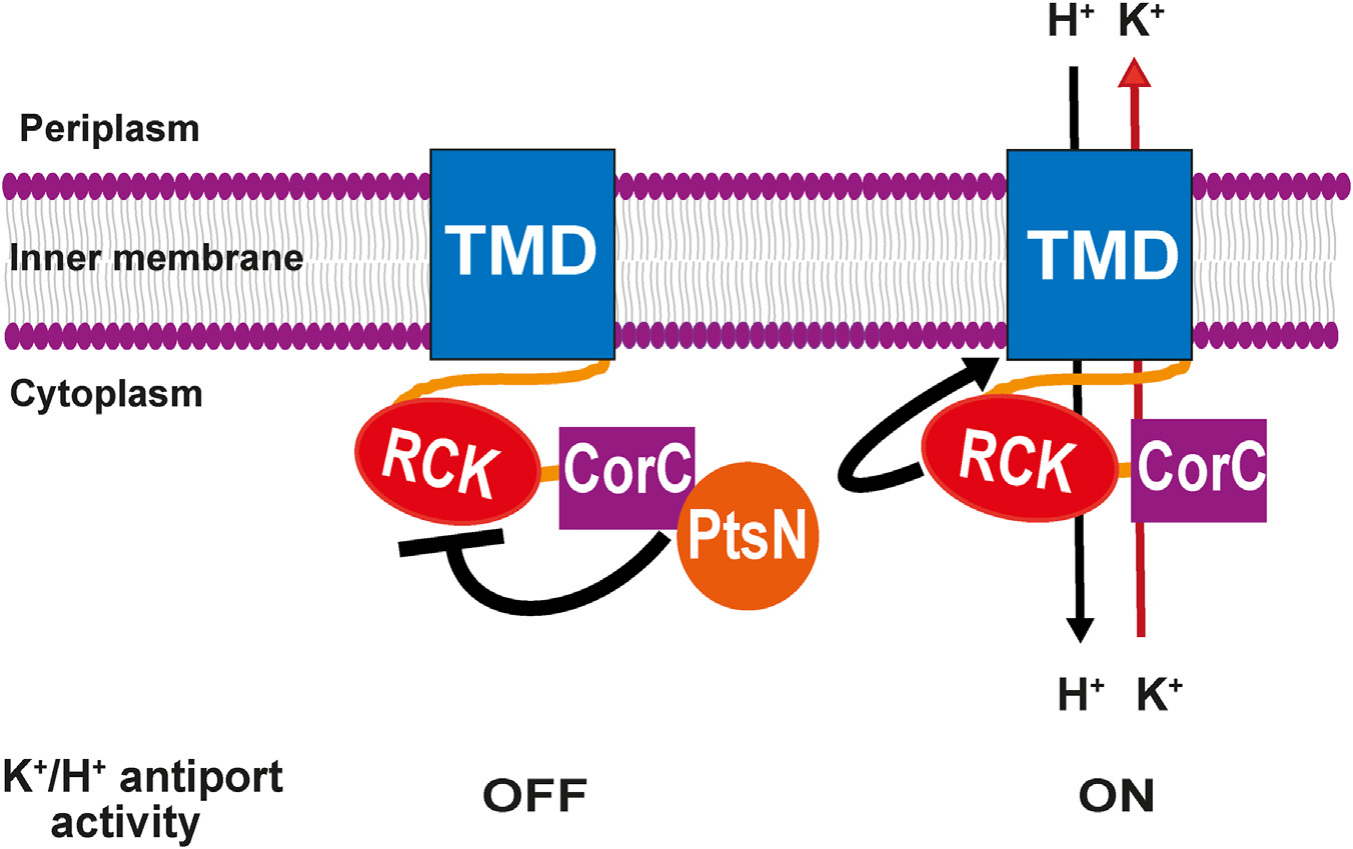
**A model for the negative regulation of the K**^**+**^**/H**^**+**^**antiport activity of YcgO, by unphospho-PtsN.** The CorC domain–bound state of unphospho-PtsN is proposed to inhibit the activation of the K^+^/H^+^ antiport function of the transmembrane domain (TMD) of YcgO by its RCK domain, leading to the silencing of YcgO activity. Activation ensues following removal of the unphospho-PtsN block. Activation and inhibition respectively are indicated by a wavy arrowhead and a wavy T symbol.

Studies reported herein were directed to identify the mechanism behind the inhibition of the K^+^/H^+^ antiport activity of YcgO by the unphosphorylated state of PtsN, unphospho-PtsN. Our studies are supportive of a model wherein the interaction of unphospho-PtsN with the CorC domain in the YcgO CTR, may antagonize the activation of YcgO by the RCK domain in the CTR, leading to inhibition of YcgO mediated K^+^ efflux (Fig. 8). It may be noted that the two domains in the CTR have been referred to as such purely based on the predictions from InterPro and the AF2 model (Fig. 1*A*) and greater validation is required for confidently assigning them as RCK and CorC. Nonetheless, the membrane topology analyses of YcgO (Figs. 1*C* and S2) overall are supportive of a two domain architecture of YcgO as predicted by the AF2 model of YcgO. The positioning of the RCK domain in the AF2 model, that is near the base of transmembrane helices likely to form the ion conducting pathway is consistent with the positioning of RCK domains in solved structures of RCK bearing K^+^ transporters and channels (50, 51, 52).

The YcgO^Con^ phenotype of YcgO_486Δ_ (Fig. 2*A*) that is associated with the inability of YcgO_486Δ_ to interact with unphospho-PtsN_His_ (Fig. 5*A*), (i) implicates the CorC domain as the site of interaction with unphospho-PtsN and (ii) supports the notion that the unphospho-PtsN:CorC interaction inhibits YcgO activity.The phenotypes of four YcgO^Con^ substitutions in the CTR (Fig. 2*A*) cohere with the observation that unphospho-PtsN targets the CorC domain of YcgO. None of the CTR YcgO^Con^ substitution bearing proteins interact proficiently with unphospho-PtsN_His_ (Fig. 5*B*). Of these, the F499Y, V542A and D545A alterations, that are in or near the CorC domain (Fig. 2, *A* and *B*), can directly perturb the interaction with unphospho-PtsN_His_, whereas the L421F substitution in the RCK domain (Fig. 2, *A* and *B*), may exert an indirect effect to perturb the interaction of unphospho-PtsN with the CorC domain. The two YcgO^Con^ substitutions in the TMD namely F38L and G273S (Fig. 2, *A* and *B*) could lead to hyperactivated YcgO mediated K^+^ efflux, thus overriding the negative influence of unphospho-PtsN. Given this, it is possible to accommodate why YcgO_G273S_ can still interact with unphospho-PtsN_His_. However, it is not clear why YcgO_F38L_ does not interact with unphospho-PtsN_His_ (Fig. 5*B*).

The phenotypes of the YcgO^Con^ substitution proteins in the CTR, indicate that relief from the unphospho-PtsN mediated block of YcgO may be accompanied by activation of YcgO. They also suggest that this feature is resident in the CTR and is latent in the presence of unphospho-PtsN. The RCK domain in the CTR can be considered to mediate activation of YcgO. The YcgO impairing effect of the L421S, and P438Q substitutions (Fig. 3, *A* and *B*) suggests that the RCK domain in the CTR may activate YcgO. The L421S, and P438Q substitutions lie in the RCK domain and the L376Q substitution in the terminal TMS, may indirectly affect RCK domain function perhaps by affecting its positioning relative to the TMD. The molecular mechanism by which the unphospho-PtsN bound CorC may inhibit the RCK mediated activation of YcgO remains to be determined.

A common yet enigmatic feature of all the YcgO^Con^ proteins is that their expression yielded the K^+^ limited growth phenotype (K^L^) only in the absence of the Trk K^+^ uptake transporter, which was not the case with the Δ*ptsN* mutant (Figs. S6 and S7). Since absence of PtsN led to a larger reduction in cellular K^+^ content in comparison to that caused by expression of the YcgO^Con^ proteins (Fig. 2*C*), it is possible that an active Trk transporter can compensate for the K^+^ loss caused by expression of the YcgO^Con^ proteins, but not by the absence of PtsN. Alternatively, PtsN, possibly unphospho-PtsN, may be required for optimal K^+^ uptake by the Trk transporter.

Prior studies have shown that *in vivo* phospho-PtsN is the predominant PtsN species (39, 40). Low amounts of unphospho-PtsN are nonetheless present, generated by the activity of the phosphohistidine phosphatase SixA that dephosphorylates PtsO, the phospho-donor of PtsN (23, 24). From a physiological viewpoint one can come to terms with the much higher interaction proficiency of unphospho-PtsN with YcgO as compared to phospho-PtsN (Fig. 4). The physiological basis for why unphospho-PtsN inhibits YcgO is not clear. It has been suggested that YcgO mediated K^+^/H^+^ antiport (Fig. 7) may represent a means of adaptation to certain stress(s) (15) which by impacting the PtsP-PtsO-PtsN phosphorelay can reduce the levels of unphospho-PtsN effecting the adaptive response *via* K^+^ efflux. If such a mechanism were to operate, it would require the conversion of a very low amount of unphospho-PtsN to phospho-PtsN, to effect a rapid adaptive response and the SixA generated unphospho-PtsN mediated inhibition of YcgO would ensue once the stress recedes.

Parallels can be drawn between YcgO and the electrophile activated K^+^ efflux protein KefC. Apart from functioning as K^+^/H^+^ antiporter, the overall membrane topology of KefC is identical to that of YcgO, that is N_OUT_-C_IN_ (53). KefC bears 13 TMSs (53) and the same number of TMSs are evident in the AF2 prediction of YcgO (Fig. 1*A*). In contrast, the Na^+^/H^+^ antiporter NhaA is organized in an N_IN_-C_IN_ 12 TMS configuration in the cytoplasmic membrane (54). Furthermore, both KefC and YcgO bear cytoplasmic CTRs with an embedded RCK domain. However, the RCK domain in KefC confers transporter regulation by a mechanism distinct to the one proposed for YcgO. For the homodimeric KefC, distinct conformational transitions caused by the inhibiting GSH and the activating GSH-conjugate, of the apposed pair of RCK domains contributed by each monomer, are thought to mediate their divergent effects on K^+^ flux through KefC (53, 55, 56). In summary, we have obtained insights into the mechanism that leads to silencing of the K^+^/H^+^ antiporter YcgO, by unphospho-PtsN. Elucidating the details on the unphospho-PtsN:YcgO complex and its impact on RCK-mediated YcgO regulation would constitute our future endeavours.

## Experimental procedures

### Bacterial strains, growth media, plasmids and recombinant DNA procedures

All bacterial strains used in this study are derivatives of *E. coli* K-12 and are described in Table S1 of the supporting information. BL21 (DE3) (57) and C41 (DE3) (58) *E. coli* B derivatives were used in some protein purification procedures. P1 transduction (59) was used for strain construction. Recombineering (60) was used in some instances of strain constructions and to append DNA sequences encoding 3× FLAG tags to chromosomal *ycgO* and its derivatives (SI methods, (61)). The amino acid numbering employed for proteins of this study refers to the natural numbering and the contribution of the appended tags is ignored. Standard molecular biology procedures for cloning PCR and overlap extension PCR were employed (62). Error prone PCR was used to isolate some mutant *ycgO* genes described in this study and the methodology is as per reference (63). Deletion insertion mutations of some genes were sourced from the Keio collection (64) and details on these are described in Table S1. Plasmids and oligonucleotide primers of this study are listed in Tables S2 and S3 respectively. Plasmids used are derivatives of the plasmids pTrc99A (65), pMU575 (37), pBAD24 and pBAD33 (46). LB agar/broth and KML agar/broth which is a modified LB medium in which NaCl is substituted by KCl (66), was used for the propagation of some strains. Antibiotics, the *lac* inducer isopropyl β-D-thiogalactoside (IPTG) and L-arabinose (Ara) the inducer of the P_*ara*_ promoter were used at appropriate concentrations and the growth temperature of bacterial strains was 37 °C. Two minimal media described in reference (67) were used to assess the growth phenotypes displayed in the figures. They are 115 mM K^+^ phosphate buffered medium and 115 mM Na^+^ phosphate buffered medium, each of pH 7.2. In this study K_1_ medium of a [K^+^]_e_ of 1 mM is 115 mM Na^+^ phosphate medium containing 1 mM KCl. K_115_ medium has a [K^+^]_e_ of 115 mM and represents 115 mM K^+^ phosphate buffered medium. The abovementioned minimal media were supplemented with glucose (0.2%), MgSO_4_ (1 mM) and supplemented with agar when required.

### Spot tests of growth phenotypes

Growth phenotypes of strains used in this study were assessed using spot tests on indicated agar plates. 40 μl of an overnight grown culture was transferred to an Eppendorf containing 960 μl of K_1_ medium from where ten-fold serial dilutions were prepared in K_1_ medium and 5.5 μl of each dilution was spotted on the surface of appropriate agar plates. For cultures cultivated in LB or KML broth, 1 ml of the cultures were washed twice in K_1_ medium suspended in 1 ml of K_1_ medium and processed for spotting as above. Media used for maintaining and cultivating strains depicted in all figures in this study are described in SI methods.

### Determination of cellular K^+^ content

Strains cultivated in K_1_ medium till an *A*_*600*_ of 0.3 to 0.6, were pelleted and resuspended in 1 ml of K_115_ medium. The Δ*ptsN* and YcgO^Con^ mutation bearing derivatives of the parent were inoculated to an initial *A*_*600*_ of 0.2 whereas the parent and the Δ*ptsN* Δ*ycgO*::*cat* double mutant were inoculated at an initial *A*_*600*_ of 0.05 in 10 ml of K_115_ medium and incubated at 37 °C for 3 h. Appropriate volumes of culture of each strain was centrifuged through a 0.2 ml layer silicone oil, prepared by mixing equal volumes of AP20 and AP100 silicone oils. The upper aqueous layer was aspirated by pipetting and residual K^+^ was removed by washing with Milli-Q water 5 times. The silicone oil layer was removed and the cell pellet was resuspended in 1 ml of a solution containing 5% nitric acid and 0.2% CsCl_2_ (solution A) and kept overnight at room temperature. Samples were boiled at 99 °C for 15 min. Cell debris was removed by centrifugation at 13,000 rpm for 5 min and 950 μl of the supernatant was transferred to new tubes and the volume was adjusted up to 5 ml with solution A. K^+^ in these samples was quantified on the iCE 3000 atomic absorption spectrophotometer. K^+^ was measured in conjunction with appropriate standards prepared in solution A. K^+^ content was expressed as nmol K^+^/*A*_*600*_ of the culture at the time of harvest. Statistical analysis of the measurements were performed using the Prism 10 suite in GraphPad.

### Protein purification

Ni-NTA affinity chromatography was used for the purification of proteins described in this study. Culture conditions detailing gene induction regimens with IPTG and NaCl (68) and purification procedures are described in SI methods as are circular dichroism and LC-ESI-MS procedures on some purified proteins. The estimations of secondary structures were carried out as described in reference (69). Proteins concentrations were measured using the Bradford method.

### Immunoblotting

3× FLAG tagged versions of YcgO and 6× His tagged versions of PtsN were detected using anti-FLAG and anti-His antibodies following separation of proteins on SDS PAGE gels. Procedure for immunoblotting and preparation of cell extracts for immunoblotting are given in SI methods. Representative images for immunoblotting performed twice are displayed. Immunoblots were developed using the ECL kit (GE Healthcare) and visualized on an ImageQuant LAS500 Imaging system. Immunoblot processing is described in the methods section of supporting information.

### Substituted cysteine (Cys) accessibility

A plasmid pHYD6507, encoding a Cys-less version of YcgO_FH_, YcgO_CL_ was constructed. YcgO_FH_ represents YcgO bearing a 3× FLAG and a 6× His tag appended to its carboxyl-terminus, after its penultimate amino acid. By site directed mutagenesis multiple mono-Cys derivatives of YcgO_CL_ were constructed. The procedure of substituted cysteine (Cys) accessibility (35, 36) was employed to obtain topological information on Cys residues introduced in YcgO_CL_. The procedure followed is identical to that described in reference 70 and is described in SI methods.

### Co-purification procedures for detecting PtsN-YcgO interaction

Strains expressing C-terminally 3× FLAG tagged YcgO and other 3× FLAG tagged proteins, were cultivated at 37 °C in LB broth to an *A*_*600*_ ranging from 2.5 to 3.0. Cells were collected by centrifugation at 4˚C and the cell pellet was washed with buffer A (20 mM Tris-Cl pH 8.0, 200 mM NaCl, 10% glycerol and 5 mM β-mercaptoethanol [BME]). Cells were resuspended in 7 ml of buffer A containing 1 mg/ml lysozyme and 1 mM PMSF and lysed with the One Shot cell disrupter Constant Systems. Unlysed cells and cell debris were removed by centrifugation at 18,000 rpm/45 min at 4 °C and 10 mM imidazole and 3 mM dodecyl-beta-D-maltoside (DDM) were added to the lysate and kept at 4 °C for 4 h for membrane protein solubilisation. *A*_*600*_ 50 equivalent cell lysates for cells expressing 3× FLAG tagged YcgO, and its derivatives and 3× FLAG tagged DsbB were transferred into a 5 ml Eppendorf tube. For cell culture expressing YcgO_486Δ_ a lysate of *A*_*600*_ equivalent of 75 was used. Twenty-five μg of C-terminally hexahistidine tagged purified proteins (unphospho-PtsN_His_, phospho-PtsN_His_ and NusG_His_) were added to DDM solubilized lysates. 100 μl aliquots were removed from lysates, serving as input fraction, prior to addition of Ni-NTA beads. 100 μl Ni-NTA beads were added to the lysates and the lysates were incubated at 4 °C on a rotary mixer for 2.5 h. Lysates were centrifuged and beads were washed with 30 bead volumes of buffer A containing 3 mM DDM, 5 mM BME, and 25 mM imidazole and washed again with 50 bead volumes of the same buffer containing 50 mM imidazole. Protein elution was performed using 100 μl of the aforementioned buffer containing 300 mM imidazole. 20 μl of the saved input samples were loaded on 12% SDS-PAGE gels. For the elution samples, 20 to 30 μl volumes adjusted for the intensity of the western signal for the hexahistidine tagged PtsN (or NusG) between eluates, were loaded. Input and elution samples were separately processed for immunodetection with anti-FLAG and anti-His antibodies following transfer of the electrophoresed samples to PVDF membrane. Furthermore, immunodetection with anti-FLAG and anti-His antibodies and blot processing are described in the methods section of supporting information.

### Fluorescence detection size exclusion chromatography (FSEC)

To test the interaction between YcgO_FH_ and unphospho-PtsN_His_ *in vitro*, purified YcgO_FH_ and unphospho-PtsN_His_ were mixed in molar ratios of 1:3 or 1:5 and run through FSEC, which uses an autosampler for injecting samples into the size exclusion column. This ensures that shifts in the observed elution profile are independent of injection volume inaccuracies. Approximately 0.1 mg/ml of YcgO_FH_ was taken per assay, and to assess its shift in elution volume, equal amounts of YcgO and unphospho-PtsN_His_ were also run separately. Tyrosine fluorescence (λex/λem = 275 nm/305 nm) was used to track protein elution, as PtsN_His_ does not possess tryptophan residues. 20 mM HEPES pH 7.0, 100 mM KCl, 100 mM NaCl and 0.1 mM LMNG (lauryl maltose neopentyl glycol) was used as the mobile phase. The assay was performed twice, with a new batch of protein each time. A Shimadzu RF-20A HPLC system was used at room temperature for this assay. A variation of the procedure for purification of unphospho-PtsN_His_ was used that is described in the methods section of supporting information.

### Detection of YcgO mediated K^+^/H^+^ antiport activity in everted vesicles

Everted vesicles were prepared from the strain GJ22224 bearing the plasmid pHYD6578, and the vector pBAD24. The procedure for their preparation is presented in SI methods. For each assay, one aliquot of vesicles was thawed at room temperature right before the start of the assay. The vesicles were resuspended in 140 mM choline chloride, 5 mM MgCl_2_, 10% (v/v) glycerol and 50 mM Bis-Tris propane-HCl, titrated to pH 7.0. A final concentration of 4 μM ACMA was added to the suspension and its fluorescence was traced at λex/λem = 409 nm/474 nm using Fluoromax-3 (Horiba) fluorescence spectrophotometer with continuous stirring. Once the fluorescence stabilized, 250 μM ATP was added to the suspension to induce formation of the proton gradient. A final concentration of 5 to 10 mM potassium or sodium (as chloride salt) was added to assess proton extrusion against the two cations. The reaction was finally quenched with the addition of 20 mM ammonium chloride. The final quenching is not depicted in the corresponding figure. Each kinetic measurement spanned 800 to 900 s. Vesicles sets were independently made twice, and multiple within batch replicates were performed for vesicles bearing vector or overexpressed YcgO_FH_.

### The AlphaFold2 predicted model of YcgO

The PDB file corresponding to the UniProt ID P76007 was downloaded from https://alphafold.ebi.ac.uk/entry/P76007 and colour rendered and annotated using PyMol.

## Data availability

The main text and supporting information contain all data pertaining to this manuscript and the corresponding author Abhijit A. Sardesai, CDFD, Hyderabad can be contacted for any queries (email: abhijit@cdfd.org.in).

## Supporting information

This article contains supporting information (15, 23, 37, 39, 40, 42, 46, 57, 58, 59, 60, 61, 63, 64, 65, 66, 68, 69, 70).

## Conflict of interest

The authors declare that they have no conflicts of interest with the contents of this article.
