# Supplementary material for: Interaction of unphosphorylated PtsN with the K+/H+ antiporter YcgO inhibits its activity in Escherichia coli
Source: J Biol Chem. 2024 Dec 30;301(2):108153. doi: 10.1016/j.jbc.2024.108153 (PMC11808508; doi:10.1016/j.jbc.2024.108153)
Supplement: Supporting information [file mmc1.docx]

**Supporting information for**

**Interaction of unphosphorylated PtsN with the K^+^/H^+^ antiporter YcgO inhibits its activity in *Escherichia coli***

Yogesh Patidar^1, 2^, Arunabh Athreya^3, 4^, Ravish Sharma^1, 5^, Aravind Penmatsa^3^ and Abhijit A. Sardesai^1^*

^1^Laboratory of Molecular Microbiology and Genetics, BRIC-Centre for DNA Fingerprinting and Diagnostics, Hyderabad, India.

^2^Graduate studies Regional Centre for Biotechnology, Faridabad, India.

^3^Molecular Biophysics Unit, Indian Institute of Science, Bangalore, India.

^4^Present address: The Department of Molecular Physiology and Biological Physics, School of Medicine, University of Virginia.

^5^Present address: Department of Chemistry and Biochemistry, University of California San Diego, USA.

*Author for correspondence: abhijit@cdfd.org.in

**Supporting information includes:**

**SI Tables S1 to S3**

**SI Methods**

**SI Figures S1 to S12**

**References**

**Table S1:** List of *E. coli* K-12 strains

| **Strain** | **Genotype** |
| --- | --- |
| ^a^MG1655 | F^-^ λ^-^ *ilvG* *rfb-50* *rph-1* |
| ^b^MC4100 | Δ(*argF*-*lac*)*U169 rpsL150 relA1 spoT1 araD139 flbB5301 deoC1 ptsF25* |
| GJ1499 | MG1655 *∆lacX74* |
| ^c^JD509 | MC4100 ∆*ptsN* ∆*ycgO*::*kan* |
| GJ17829 | MG1655 *∆lacX74* ∆*trkA* ∆*kup* |
| GJ17831 | GJ17829 ∆*ptsN* |
| GJ17854 | GJ17829 *ycgO*_F_:*kan* |
| GJ18836 | MC4100 ∆*ycgO*::*kan* |
| GJ18837 | GJ17829 ∆*ycgO*::*kan* |
| GJ19262 | GJ17829 ∆*ycgO*::*cat* |
| GJ19263 | GJ17829 *ycgO*_F499YF_:*kan* |
| GJ19264 | GJ17829 *ycgO*_L421FF_:*kan* |
| GJ19265 | GJ17829 *ycgO*_V542AF_:*kan* |
| GJ19266 | GJ17829 *ycgO*_D545AF_:*kan* |
| GJ19267 | GJ17829 *ycgO*_F38LF_:*kan* |
| GJ19290 | GJ17829 *ycgO*_G273SF_:*kan* |
| GJ19291 | GJ17829 *ycgO*_486∆F_:*kan* |
| GJ19300 | GJ1499 ∆*ptsN* ∆*ptsP*::*kan* |
| GJ21012 | GJ1499 *malQ*::P*proU*T7RNAP ∆*ptsN* ∆*sixA*::*kan* |
| GJ21015 | GJ19300 ∆*ptsP* |
| GJ21016 | GJ21012 ∆*sixA* |
| GJ21019 | GJ21015 *dsbB*_F_:*kan* |
| GJ21020 | GJ21015 *ycgO*_F_:*kan* |
| GJ21021 | GJ21015 *ycgO*_L421FF_:*kan* |
| GJ21022 | GJ21015 *ycgO*_F499YF_:*kan* |
| GJ21023 | GJ21015 *ycgO*_V542AF_:*kan* |
| GJ21024 | GJ21015 *ycgO*_D545AF_:*kan* |
| GJ22211 | GJ21015 *ycgO*_F38LF_:*kan* |
| GJ22212 | GJ21015 *ycgO*_G273SF_:*kan* |
| ^1,4^GJ21069 | MC4100 ∆*kdpA ycgO*_F_:*kan* |
| ^1^GJ21070 | GJ21069 *ycgO*_F38LF_:*kan* |
| ^1^GJ21071 | GJ21069 *ycgO*_G273SF_:*kan* |
| ^1^GJ21072 | GJ21069 *ycgO*_L421FF_:*kan* |
| ^1^GJ21073 | GJ21069 *ycgO*_F499YF_:*kan* |
| ^1^GJ21074 | GJ21069 *ycgO*_V542AF_:*kan* |
| ^1^GJ21075 | GJ21069 *ycgO*_D545AF_:*kan* |
| ^2,5^GJ21076 | MC4100 ∆*kup* *ycgO*_F_:*kan* |
| ^2^GJ21077 | GJ21076 *ycgO*_F38LF_:*kan* |
| ^2^GJ21078 | GJ21076 *ycgO*_G273SF_:*kan* |
| ^2^GJ21079 | GJ21076 *ycgO*_L421FF_:*kan* |
| ^2^GJ21080 | GJ21076 *ycgO*_F499YF_:*kan* |
| ^2^GJ21081 | GJ21076 *ycgO*_V542AF_:*kan* |
| ^2^GJ21082 | GJ21076 *ycgO*_D545AF_:*kan* |
| ^3,6^GJ21083 | MC4100 ∆*trkA* *ycgO*_F_:*kan* |
| ^3^GJ21084 | GJ21083 *ycgO*_F38LF_:*kan* |
| ^3^GJ21085 | GJ21083 *ycgO*_G273SF_:*kan* |
| ^3^GJ21086 | GJ21083 *ycgO*_L421FF_:*kan* |
| ^3^GJ21087 | GJ21083 *ycgO*_F499YF_:*kan* |
| ^3^GJ21088 | GJ21083 *ycgO*_V542AF_:*kan* |
| ^3^GJ21089 | GJ21083 *ycgO*_D545AF_:*kan* |
| GJ22204 | GJ21015 *ycgO*_486∆F_:*kan* |
| GJ22206 | GJ17831 ∆*ycgO*::*cat* |
| GJ22207 | GJ17831 *ycgO*_F_:*kan* |
| GJ22224 | MC4100 ∆(*araBAD*)567 ∆*ptsN* ∆*chaA* ∆*ybaL* ∆*ycgO* ∆*kefC* ∆*kefB* ∆*nhaA* |
| ^1,4^GJ22278 | GJ21069 ∆*ptsN* |
| ^2,5^GJ22279 | GJ21076 ∆*ptsN* |
| ^3,6^GJ22280 | GJ21083 ∆*ptsN* |
| ^4^GJ22281 | GJ21069 *ycgO_486∆_*_F_:*kan* |
| ^5^GJ22282 | GJ21076 *ycgO_486∆_*_F_:*kan* |
| ^6^GJ22283 | GJ21083 *ycgO_486∆_*_F_:*kan* |

All strains used in this study are derivatives of *Escherichia coli* K-12. ^a^MG1655 and ^b^MC4100 are from laboratory stocks, ^c^described in reference 15, ^1,2,3,4,5,6^ are strains used in supplemental figures S6 and S7 (Panels A, B and C). Strains from the Keio knockout collection (64) were used to introduce the ∆*ptsN*::*kan*, ∆*ptsP*::*kan*, ∆*kdpA* ::*kan*, ∆*trkA*::*kan*, ∆*chaA*::*kan*, ∆*ybaL*::*kan*, ∆*ycgO*::*kan*, ∆*sixA*::*kan*, ∆*kefB*::*kan*, ∆*kefC*::*kan* and ∆*nhaA*::*kan* deletion insertions into appropriate strains by P1 transduction (59). The ∆*kup*::*kan* and ∆*ycgQ*::*kan* knockout mutations were constructed by recombineering (60) and the physical arrangement of these deletion insertions is identical to other deletion insertions from the Keio knockout collection. The *kan* gene in all the aforementioned deletion insertion mutations is flanked by a pair of FRT (Flippase recognition target) sites. The *kan* gene was excised following treatment with the plasmid pCP20 (60). Such mutations are indicated with “∆” symbol. The ∆*ycgO*::*cat* mutation was constructed by recombineering. The *cat* marker recombineered is without the flanking FRT sites. Instances of *kan* placed adjacent to a particular gene, have been indicated with a colon (:) symbol. Genes encoding proteins bearing 3x FLAG peptide appended to their C-termini are marked with a subscripted “F” after the corresponding gene designation. In some instances, the subscripted “F” symbol is preceded by a substitution or a deletion designation. For example, the gene *ycgO*_L421FF_, encodes YcgO protein that is 3x FLAG tagged at its C-terminus and bears the L421F substitution. In *ycgO*_486∆F_, the encoded 3x FLAG tagged YcgO lacks the C-terminal 92 amino acids.

**Table S2:** Plasmids used in this study

| **Plasmid ID** | **Plasmid Details** |
| --- | --- |
| ^a^pHYD5001 | Derivative of the plasmid ^b^pTrc99A, bearing a replacement of a unique NcoI site with an NdeI site. The pre-existing NdeI site in PTrc99A has been destroyed. |
| ^c^pHYD5006 | Derivative of the pHYD5001, capable of expressing PtsN_His6_. The corresponding gene is present in the NdeI and HindIII sites. |
| pHYD5011 | Derivative of pTrc99A capable of expressing YcgO_FH_. The corresponding gene is present in NcoI and HindIII sites. |
| pHYD6479 | Derivative of the plasmid pHYD5001 containing the *ori* and the *cat* gene from the plasmid ^d^pBAD33. |
| ^e^pHYD1852 | Derivative of pHYD5001, *kup* expression is under P*_trc_* control and *kup* is present between EcoRI and HindIII sites. |
| pHYD6023 | Derivative of the plasmid ^f^pMU575 in which the ∆*ycgQ- ycgO* chromosomal DNA sequence along with 345 bp and 157 bp of DNA sequences located upstream of *ycgQ* and downstream of *ycgO* respectively is present in the KpnI and HindIII sites. |
| pHYD6042 | Derivative of the plasmid pHYD6023 in which the *cat* cassette of ^g^pKD3 without flanking FRT sites was recombined into pHYD6023 downstream of the translational termination codon of *ycgO*. |
| pHYD6048 | Derivative of the plasmid pHYD6023 in which *ycgO* gene is replaced with the *kan* element of ^g^pKD13 without the flanking FRT sites by recombineering. |
| pHYD6067 | Derivative of pHYD6042 encoding YcgO with the F499Y amino acid substitution. |
| pHYD6069 | Derivative of pHYD6042 encoding YcgO with the G273S amino acid substitution. |
| pHYD6072 | Derivative of pHYD6042 encoding YcgO with the L421F amino acid substitution. |
| pHYD6073 | Derivative of pHYD6042 encoding YcgO with the V542A amino acid substitution. |
| pHYD6077 | Derivative of pHYD6042 encoding YcgO with the D545A amino acid substitution. |
| pHYD6522 | Derivative of pHYD6042 encoding YcgO with the F38L amino acid substitution. |
| pHYD6506 | Derivative of pHYD5011 encoding YcgO_CL_ with the natural cysteine residue at amino acid number 427. |
| pHYD6507 | Derivative of pHYD5011, encoding for YcgO_CL_ (Cysless- YcgO) that bears the C252A, C427A and C468A amino acid substitutions |
| pHYD6508 | Derivative of pHYD5011 encoding YcgO_CL_ with the natural cysteine residue at amino acid number 252. |
| pHYD6509 | Derivative of pHYD5011 encoding YcgO_CL_ with the natural cysteine residue at amino acid number 468. |
| pHYD6510 | Derivative of pHYD6507, encoding YcgO_CL_ with the A386C substitution. |
| pHYD6511 | Derivative of pHYD6507, encoding YcgO_CL_ with the A3C substitution. |
| pHYD6512 | Derivative of pHYD6507, encoding YcgO_CL_ with the A444C substitution.. |
| pHYD6513 | Derivative of pHYD6507, encoding YcgO_CL_ with the G519C substitution. |
| pHYD6514 | Derivative of pHYD6507, encoding YcgO_CL_ with the A557C substitution. |
| pHYD6515 | Derivative of pHYD6507, encoding YcgO_CL_ with the A576C substitution. |
| pHYD6516 | Derivative of pHYD6507, encoding YcgO_CL_ with the V393C substitution. |
| pHYD6517 | Derivative of pHYD6507, encoding YcgO_CL_ with the I500C substitution. |
| pHYD6518 | Derivative of pHYD6507, encoding YcgO_CL_ with the L383C substitution. |
| pHYD6520 | Derivative of pTrc99A encoding YcgO_NF_ wherein YcgO is appended with an N-terminal 3x FLAG tag appended to its second amino acid. The corresponding gene is present between NcoI and HindIII sites. |
| pHYD6521 | Derivative of pHYD6520 encoding YcgO_NFX_. The gene encoding YcgO_NF_ was modified to introduce an XhoI restriction site such the 358^th^ codon (CTG) in *ycgO* was altered to (CTC). |
| pHYD6523 | Derivative of pHYD6521, in which the DNA in *ycgO* between the XhoI and the HindIII sites was replaced with the *kan* element from pKD13 with flanking FRT sites, without altering the two sites. |
| pHYD6531 | Derivative of pHYD6507, encoding YcgO_CL_ with the L378C substitution. |
| pHYD6532 | Derivative of pHYD6521, encoding YcgO_NFX_ with the L376Q amino acid substitution. |
| pHYD6542 | Derivative of pACYCduet-1 capable of expressing PtsP and PtsO from the two different T7 promoters. *ptsP* and *ptsO* are present between NcoI-HindIII and NdeI-XhoI sites respectively |
| pHYD6547 | Derivative of the plasmid pET28b encoding CTR_His6_ (386 codons onwards)_._ Wherein YcgO CTR is appended with a hexahistidine tag at the C-terminus. The corresponding gene is present between NcoI and HindIII sites. |
| pHYD6907 | Derivative of pHYD6521, encoding YcgO_NFX_ with the P438Q amino acid substitution. |
| pHYD6909 | Derivative of pHYD6521, encoding YcgO_NFX_ with the L421S amino acid substitution. |
| pHYD6578 | Derivative of ^d^pBAD24, capable of expressing YcgO with a 3x FLAG tag appended to its C-terminus. The corresponding gene is present between the NcoI and HindIII sites. |
| pHYD6580 | Derivative of the plasmid pHYD6479, capable of expressing PtsN_His6_. The corresponding gene is placed between the NdeI and HindIII sites. |
| pHYD6581 | Derivative of pETduet-1 capable of expressing PtsP and PtsO from the two different T7 promoters. *ptsP* and *ptsO* are present between NcoI-HindIII and NdeI-XhoI sites respectively. |
| pHYD6901 | Derivative of pHYD6507, encoding YcgO_CL_ with the G76C substitution. |
| pHYD6902 | Derivative of pHYD6507, encoding YcgO_CL_ with the T169C substitution. |
| pHYD6903 | Derivative of pHYD6507, encoding YcgO_CL_ with the A368C substitution. |
| ^h^pRS119 | Derivative of pET21a encoding for NusG_His6,_ wherein The corresponding gene is present between the XhoI and HindIII sites. |

Described in ^a^15, ^b^65, ^c^15, ^d^46, ^e^15, ^f^37, ^g^60 and ^h^42. The plasmids pETDuet-1, pACYCduet-1, pET28b and pET21a are distributed by Novagen.

**Table S3:** Primers used in this study

| **Primer ID** | **Primer sequence (5'-3')** | **Purpose** |
| --- | --- | --- |
| JGAFYCGQKOF | GCATGATATACATTTCGTTTCTGTTGTCAGCAAGGAATTGCCATGATTCCGGGGATCCGTCGACC | For the construction of ∆*ycgQ* on the plasmid pHYD6023. |
| JGAFYCGQKOR | ACTTAGCCCTGAAGCGTGATTTTTTTACATTTTAAGAACAGGATGGTGTAGGCTGGAGCTGCTT | As above |
| JGAFYCGOCMF | AGGCAATTACACAAATCTATGTACAGGCTCCATCAAGGGTCGCCTACCTGTGACGGAAGA | For the construction of the *ycgO*::*cat* in pHYD6042 |
| JGAFYCGOCMR | AGCTCGAATTAATTCCCGATCTGCAGGTCGACGGATCCAAGCTTTCATCGCAGTACTGTTGTATT | As above |
| JGVDSBBF | ATTTCCCAGCCGTTTAAAGCGAAAAAACGTGATCTGTTCGGTCGCGACTACAAAGACCATGACGG | For the construction of *dsbB*_F_ on the chromosome. |
| JGVDSBBR | TAGCGGCAGGAAAAAAGCGCTCCCGCAGGAGCGCCGAATGGATTACATATGAATATCCTCCTTAG | As above |
| JGAFYCGOEPFP | TCTTAAAATGTAAAAAAATCACGCTTCAGGGCT | For performing error-prone PCR of the insert on the plasmid pHYD6042 |
| JGSQPMU575FP | TCCCCACATCACCAGCAA | As above |
| JGAFYCGODELFP | TAGGGTTTATATAAGAGACAGCGTAATCAGGAGTAACCGACCGGAGCGCTTTTGAAGCTCAC | For the replacement of *ycgO* with *kan* cassette on the plasmid pHYD6048 |
| JGAFYCGODELRP | TCGAATTAATTCCCGATCTGCAGGTCGACGGATCCAAGCTTTTAGAAGAACTCGTCAAGA | As above |
|  |  |  |
| JGVYCGOF1 (1) | GTGTTGAAGATTGGCGTACGGGTGGCTGAAGAGGAAGCTGAATCTGACTACAAAGACCATGACGG | For the construction of *ycgO*_F_ on the chromosome. |
| JGAFYCGO 486FLAGFP (2) | CGGGAACGCGATCTTCCGGCGCTCGGTAAACTGTTCAGCCAGTCGGACTACAAAGACCATGACGG | For the construction of *ycgO*_486∆F_ on the chromosome. |
| JGVYCGOF2 | TTCAGCAAAGCATCGCAACCGGCGATAGATGCTTCATTAAATTTACATATGAATATCCTCCTTAG | Paired with (1), (2) for PCR and for the construction of chromosomally encoded 3x FLAG tagged derivatives of YcgO. |
| JGAFYCGOCMWSFP | GTAGGGTTTATATAAGAGACAGCGTAATCAGGAGTAACCGACCGCGCCTACCTGT GACGGAAGA | For the construction of the chromosomal *ycgO*::*cat* mutation. |
| JGAFYCGOCMWSRP | GTTCAGCAAAGCATCGCAACCGGCGATAGATGCTTCATTAAATTCATCGCAGTACTGTTGTA TT | As above |
| JGSQPMU575GalKRP | CAGAGATTGTGTTTTTTCTTTCAG | For performing overlap extension PCR for the construction of plasmids expressing multiple YcgO^Con^ substituted derivatives of pHYD6042. |
| JGAFG273SF | ATCCTGCAAAATTTCGACAGCCTCGCCTGGCTGGCGCAA | For the construction of the plasmid pHYD6069 and chromosomally encoded YcgO bearing the G273S substitution. |
| JGAFG273SR | TTGCGCCAGCCAGGCGAGGCTGTCGAAATTTTGCAGGAT | As above |
| JGAFF499YF | ACGCTTCTTTGGTGACTACATTCTCGAAGCCAGCGCCAA | For the construction of the plasmid pHYD6067 and chromosomally encoded YcgO bearing the F499Y substitution. |
| JGAFF499YR | TTGGCGCTGGCTTCGAGAATGTAGTCACCAAAGAAGCGT | As above |
| JGAFL421FF | AGCAGTTTGTTTATCAATTTAGTGCCGATAAATGGTGCGT | For the construction of the plasmid pHYD6072 and chromosomally encoded YcgO bearing the L421F substitution. |
| JGAFL421FR | ACGCACCATTTATCGGCACTAAATTGATAAACAAACTGCT | As above |
| JGAFV542AF | TGTTAGGCGCGGCACCGGCTGTCGGTGACCAGGTAGA | For the construction of the plasmid pHYD6073 and chromosomally encoded YcgO bearing the V542A substitution. |
| JGAFV542AR | TCTACCTGGTCACCGACAGCCGGTGCCGCGCCTAACA | As above |
| JGAFD545AFP | AGGCGCGGCACCGGTTGTCGGTGCCCAGGTAGAGTTTGCCGGGATG | For the construction of the plasmid pHYD6077 and chromosomally encoded YcgO bearing the D545A substitution. |
| JGAFD545ARP | CATCCCGGCAAACTCTACCTGGGCACCGACAACCGGTGCCGCGCCTA | As above |
| JGAFYNFFP1 | ATAAAGATCATGACATCGACTACAAGGATGACGATGACAAGGATGCCACAACAATAATT | For the construction of the plasmid pHYD6520 |
| JGAFYNFLAGFP | ATACTGACTCAGCCATGGACTACAAAGACCATGACGGTGATTATAAAGATCATGACATCGAC | As above |
| JGKYCGOR | AGATGCTTCAAAGCTTTTAAGATTCAGCTTCCTC | As above |
| JGAFL358 XHOIFP | GATGATGGCGGGGCTCGAGAATGCACGTCTGTTCTTTAATGT | For the construction of the plasmid pHYD6521 |
| JGAFL358 XHOIRP | ACATTAAAGAACAGACGTGCATTCTCGAGCCCCGCCATCATC | As above |
| JGAFPKD13XHOIF | ATCGCGATACCTCGAGATTCCGGGGATCCGTCGACC | For the construction of the plasmid pHYD6523 |
| JGAFPKD13HINDIIIR | ATCGTCGATAAGCTTTGTAGGCTGGAGCTGCTTCG | As above |
| JGKYCGOS2 | AAGCGATCTGCTGCCTATTG | For performing error prone PCR of the insert on the plasmid pHYD6521. |
| JGSQPtrcRP | TGGGACCACCGCGCTA | As above |
| JGAFPTRCUSF | AGTGGGATACGACGATACCGAAGACA | For performing overlap extension PCR for the construction of plasmids expressing multiple mono-cysteine (Cys) substituted derivatives of YcgO_CL_. |
| JGAFPTRCUSR | AGGGTTATTGTCTCATGAGCGGA | As above |
| JGSQPtrcFP | CGACATCATAACGGTTCTGG | As above |
| JGAFC252AFP | TTCTGGCGGTGTATCTGGCGGGTTTTCTGCTGGGTAATC | For the construction of the plasmid pHYD6507 |
| JGAFC252ARP | GATTACCCAGCAGAAAACCCGCCAGATACACCGCCAGAA | As above |
| JGADCLYIF2 | CGATAAATGGGCTGTGGGCGCGGCA | As above |
| JGADCLYIR2 | CGCGCCCACAGCCCATTTATCGGCACTCAATT | As above |
| JGADCLYIF3 | GCGATGTGTTGGCGGTAATTGGTCGGGAAC | As above |
| JGADCLYIR3 | GTTCCCGACCAATTACCGCCAACACATCGC | As above |
| JGAFYCGOA3CNCOIF | CAGAAACAGACCATGGATTGCACAACAATAATTAGCCT | For the construction of the plasmid pHYD6511 |
| JGAFYCGOL383CF | CTTGCAGGGAACATCATGCTCGTGGGCGGCTAAAA | For the construction of the plasmid pHYD6518 |
| JGAFYCGOL383CR | TTTTAGCCGCCCACGAGCATGATGTTCCCTGCAAG | As above |
| JGAFYCGOA386CF | GAACATCACTCTCGTGGTGCGCTAAAAAAGCCAAAGTG | For the construction of the plasmid pHYD6510 |
| JGAFYCGOA386CR | ACTTTGGCTTTTTTAGCGCACCACGAGAGTGATGTTCC | As above |
| JGAFYCGOV393CF | CTAAAAAAGCCAAAGTGTGCGTTCCGCCAGTGGGAC | For the construction of the plasmid pHYD6516 |
| JGAFYCGOV393CR | GTCCCACTGGCGGAACGCACACTTTGGCTTTTTTAG | As above |
| JGAFYCGOA444CR | GTTATCACGAAACAGTGCGCAAATACGCGTCTCTTTTGG | For the construction of the plasmid pHYD6512 |
| JGAFYCGOA444CF | CCAAAAGAGACGCGTATTTGCGCACTGTTTCGTGATAAC | As above |
| JGAFYCGOI500CF | CTTCTTTGGTGACTTCTGCCTCGAAGCCAGCGCCA | For the construction of the plasmid pHYD6517 |
| JGAFYCGOI500CR | TGGCGCTGGCTTCGAGGCAGAAGTCACCAAAGAAG | As above |
| JGAFYCGOG519CF | ATATATGGCCTGGAAGACTGCCGAGAATATCGTGATAAGC | For the construction of the plasmid pHYD6513 |
| JGAFYCGOG519CR | GCTTATCACGATATTCTCGGCAGTCTTCCAGGCCATATAT | As above |
| JGAFYCGOA557CF | GATGATCTGGACGGTGTGCGAGAAAGAAGATAATG | For the construction of the plasmid pHYD6514 |
| JGAFYCGOA557CR | CATTATCTTCTTTCTCGCACACCGTCCAGATCATC | As above |
| JGAFYCGOA576CF | GGGTGGCTGAAGAGGAATGCGAATCTGACTACAAAG | For the construction of the plasmid pHYD6515 |
| JGAFYCGOA576CR | CTTTGTAGTCAGATTCGCATTCCTCTTCAGCCACCC | As above |
| JGAFL378CFP | CTGGTTTCACTGCTCTGCCAGGGAACATCACTCTC | For the construction of the plasmid pHYD65231 |
| JGAFL378CRP | GAGAGTGATGTTCCCTGGCAGAGCAGTGAAACCAG | As above |
| JGKYCGOF | TAACCGACCGCCATGGATGCCACAACAATAATTAG | For the construction of pHYD5011, pHYD6578 and other YcgO derivative plasmid. |
| JGVDSBBFHIS4 | TAACCCAAGCTTCTAATGGTGATGATGGTGGTGTTTATCGTCGTCATCTTTGTAGTCG | For the construction of the plasmid pHYD5011. |
| JGAFFLAGH3RP | GCTCACTGCAGAAGCTTCTATTTATCGTCGTCATCTTTGTAGTC | For the construction of pHYD6578. |
| JGAFL376QFP | TGGTTCTGGTTTCACAGCTCTTGCAGGGAACATC | For the construction of the plasmid pHYD6532 |
| JGAFL376QRP | GATGTTCCCTGCAAGAGCTGTGAAACCAGAACCA | As above |
| JGADPTSONDF | GGAAAAACGTAACATATGACCGTCAAGCAAACTGT | For the construction of the plasmid pHYD6581 and pHYD6542. |
| JGADPTSOXHR | ATGTCAAAGTGACTCGAGTTAATCTTCATCAAAACCAGA | As above |
| JGADPTSPH3R | TATGTATATGAAAGCTTCTATAACCCTCCGCGAATCA | As above |
| JGADPTSPNCF | AGTCACGCCAACCATGGTCACTCGCCTGCGCGAAATAGT | As above |
| JGAFY386NCOIFP | GATCAATTCACCATGGCGGCTAAAAAAGCCAAAGTG | For the construction of the plasmid pHYD6547. |
| JGAFYcgOHisRP | TTCATCTCAGAAGCTTTTAATGGTGATGATGGTGGTGAGATTCAGCTTCCTCTTCAG | As above |
| JGAFYcgOG76CFP | TGGCGATTATTTTGCTCGACTGCGGGATGCGCACTCAGGC | For the construction of the plasmid pHYD6901. |
| JGAFYCGOG76CRP | GCCTGAGTGCGCATCCCGCAGTCGAGCAAAATAATCGCCA | As above |
| JGAFYcgOT169CFP | CAATGGCGGTCTTTCTGTGCATTACCCTAATTGCGATGATC | For the construction of the plasmid pHYD6902. |
| JGAFYcgOT169CRP | CATCGCAATTAGGGTAATGCACAGAAAGACCGCCATTGGA | As above |
| JGAFA368CFP | CGTCTGTTCTTTAATGTCTGCTTCTTTGTGGTTCTGGT | For the construction of the plasmid pHYD6903. |
| JGAFA368CRP | ACCAGAACCACAAAGAAGCAGACATTAAAGAACAGACG | As above |

Primers for sequence verification of the described plasmids and recombineering constructs are not listed.

**Methods**

**Media used for strain propagation**

**Figure 2**

For strains in panels A and C the parent and ∆*ptsN* ∆*ycgO* (∆*ycgO*::*cat*) were maintained on KML agar. The ∆*ptsN* derivative and parent expressing the indicated YcgO^Con^ amino acid substitutions and YcgO_486∆_ were obtained and maintained on K_1_ agar. For spot tests, the parent, its ∆*ptsN* derivative and the YcgO^Con^ expressing strains were cultivated in K_1_ medium prior to spotting.

**Figure 3**

The strain GJ22206 was obtained on K_1_ agar and maintained on both K_1_ and KML medium/agar during experiments. GJ22206 was cultivated in KML broth and transformed with plasmids expressing the indicated YcgO proteins. Transformants were selected and maintained on K_1_ agar. For spot tests and immunoblotting (panels A, C), strains were cultivated in K_1_ medium.

**Figure S1**

The strain JD509 was transformed with the vector and plasmids expressing the indicated YcgO proteins and transformants were selected on LB agar. For spot tests transformants were cultivated in LB broth and cultures were washed twice with 1 ml of K_1_ medium and spotted on K_1_ and K_115_ agar plates.

**Figure S2 and 1C**

The strain GJ18836 bearing plasmids expressing the indicated mono-Cys derivatives of YcgO_CL_ were maintained on LB agar and for Cys accessibility experiments, strains were cultivated in LB broth.

**Figure S4**

Strains for immunoblotting in panels A and B were cultivated in K_1_ medium.

**Figure S5**

All employed strains were transformed with vector and pHYD1852 and transformants were selected and maintained on K_1_ agar. For spot tests strains were cultivated in K_1_ medium.

**Figures S6 and S7**

For figures S6A, B and S7 A, B, the parent, its ∆*ptsN* derivative and parent expressing indicated YcgO^Con^ proteins were constructed and maintained on KML broth/agar. For spot tests, strains were cultivated in K_1_ medium. In figures S6C and S7C**,** the parent, its ∆*ptsN* derivative and parent expressing the indicated YcgO^Con^ proteins were constructed and maintained on K_1_ agar and cultivated in K_1_ medium for spot tests.

**Substituted cysteine (Cys) accessibility**

Mid-exponential phase cultures of the strain GJ18836, bearing plasmids encoding mono-Cys substituted derivatives of YcgO_CL_ were cultivated in LB broth at 37°C with appropriate antibiotic selection, were harvested and washed with phosphate buffered saline (PBS) pH 7.4. Harvested cells were suspended in 200 μl of PBS, at an *A*_600_ of 2. Resuspended cells were distributed in four 50 μl aliquots in four microfuge tubes. Two aliquots were treated separately with the cysteine sulfhydryl blockers NEM (*N*-ethylmaleimide) and MTSES (sodium (2-sulfonatoethyl) methanethiosulfonate), each at a final concentration of 5 mM. The four tubes were placed at room temperature for 60 minutes in dark and subjected to intermittent mild agitation. Cells were washed twice with 1 ml of PBS and resuspended in 50 μl of lysis buffer (15 mM Tris-HCl, pH 7.4, 1% SDS, 6 M urea). Of the four aliquots, the two blocked samples and one sample not exposed to either NEM or MTSES were labelled with Mal-PEG (methoxypolyethylene glycol maleimide, molecular weight 5000 Da) present at 5 mM, and the last sample was treated with DMSO (solvent control). Samples were kept in dark at room temperature for 60 minutes and intermittently agitated mildly. Further sample processing was as per reference 70. Following SDS PAGE, the gels were processed for immunoblotting with anti-FLAG antibody.

**Isolation of mutant *ycgO* derivatives yielding the YcgO^Con^ phenotype.**

To isolate missense mutations in *ycgO* that lead to the YcgO^Con^ phenotype, a plasmid pHYD6023, was constructed. This plasmid is a derivative of the single copy promoter probe vector pMU575 (37) that contains a segment of DNA bearing the ∆*ycgQ* *ycgO* configuration flanked with 345 and 156 bp of DNA located upstream and downstream respectively to the *ycgQ ycgO* gene pair, placed in the KpnI and HindIII sites. The ∆*ycgQ* deletion contains an in frame FRT site. Immediately after the FRT scar sequence we noted an insertion of a cytosine residue. A chloramphenicol (Cm) resistance *cat* cassette, sourced from the plasmid pKD3 (60) without the flanking FRT sites, was placed immediately upstream of the HindIII site that is located downstream to the *ycgO* translation termination codon in pHYD6023, via recombineering (60) generating the plasmid pHYD6042. *ycgO* in pHYD6023 was replaced with a kanamycin (Kan) resistance cassette lacking the flanking FRT sites, sourced from the plasmid pKD13 (60), by recombineering, generating the plasmid pHYD6048. pHYD6042 was used as a template to amplify *ycgO* and the adjoining *cat* cassette using the primer pair JGAFYCGOEPFP and JGSQPMU575FP, by error prone PCR as per the method described in reference 63. These primers also carried terminal (5′) homologies, permitting recombineering of the amplified PCR products into pHYD6048. The error prone PCR product library was recombineered into MG1655 bearing the plasmids pKD46 (60) and pHYD6048. Recombinants were selected on LB agar supplemented with Cm and trimethoprim (Tp) that selects for pHYD6048. 400 Cm and Tp resistant recombinants were pooled and plasmid was isolated from this pool. This plasmid preparation was introduced into GJ18837 and transformants were obtained on K_1_ agar containing Tp and Cm. 700 plasmid transformants were tested for growth on K_1_ and K_115_ agar. 13 transformants were found, that display a growth phenotype identical to the ∆*ptsN* mutant, which is the ability and the inability to grow in K_1_ and K_115_ agar respectively (15). This phenotype is designated as K^L^ and the basis for this designation is explained in the text of this manuscript. The K^L^ yielded by the aforementioned 13 transformants is designated as the YcgO^Con^ phenotype. All the 13 transformants were Cm resistant and Kan sensitive indicative of faithful recombineering. Plasmids with the YcgO^Con^ phenotype were sequenced to identify the spectrum of base substitutions causal to their phenotype. Lastly, via site directed mutagenesis single codon alterations leading to the YcgO^Con^ phenotype were identified. Strains hosting the YcgO^Con^ phenotype conferring plasmids were, maintained and propagated in K_1_ medium with the appropriate antibiotic selection, during the step of phenotype testing.

**Recombineering procedures:**

The procedure of recombineering (60) was used to construct genes encoding the various YcgO proteins that are expressed from their native chromosomal location. All these genes encode YcgO proteins bearing C-terminally abutted 3x FLAG tag. A *dsbB* gene encoding a protein with a similarly placed 3x FLAG tag was also constructed by recombineering. The strain expressing the wild type 3x FLAG tagged YcgO has been described earlier (15). The external (5′) regions of the primers for recombineering contained 40-45 nucleotide homologies to the chromosomal DNA whereas the internal regions bore homology to a specific region of the plasmid pSUB11 (61), containing the nucleotide sequence encoding the 3x FLAG epitope and the *kan* gene, flanked by a pair of FRT sites. PCR products were electroporated into MG1655 in most cases, bearing the plasmid pKD46 with the host strain cultivated as described in reference 60. Kan resistant recombinants were isolated on Kan supplemented LB agar plates and the corresponding genes were transferred to other strains by P1 transduction (59). Strains bearing genes encoding epitope tagged proteins in the MG1655 background are not listed in Table S1. MG1655 bearing the ∆*ycgO*::*cat* mutation was constructed by recombineering. In this strain the sequence of *ycgO* between the translational initiation and termination codons is replaced with the *cat* cassette from the plasmid pKD3 without the flanking FRT sites. To construct chromosomal derivatives of *ycgO,* expressing YcgO^Con^ proteins with an appended 3x FLAG tag, genes encoding these mutant proteins were linked to the 3x FLAG *kan* cassette of pSUB11 by overlap extension PCR, and the obtained product was recombineered into MG1655 bearing the ∆*ycgO*::*cat* mutation. All Kan resistant recombinants obtained were rendered Cm sensitive indicative of faithful recombineering events. These genes were introduced into other strains by P1 transduction. Primers used for recombineering are listed in Table S3.

**Identification of YcgO impairing amino acid substitutions in the CTR**

A plasmid pHYD6520 with a *ycgO* gene, *ycgO*_NF_, placed under the expression control of the P*_trc_* promoter was constructed by PCR. *ycgO*_NF_, encodes the protein YcgO_NF_ bearing an N-terminal 3x FLAG tag and the last amino acid of the FLAG epitope is linked to the second amino acid of YcgO. Via site-directed mutagenesis a derivative of pHYD6520, pHYD6521, was constructed wherein the codon encoding the 358^th^ amino acid leucine, CTG of *ycgO* was altered to CTC. This leads to a synonymous codon alteration in *ycgO*_NF_. The obtained gene with a unique internal XhoI site and the encoded protein are designated *ycgO*_NFX_ and YcgO_NFX_ respectively. The DNA within the XhoI HindIII segment of *ycgO*_NFX_ was replaced with the FRT flanked Kan resistance cassette (*kan*) of the plasmid pKD13, obtained plasmid designated as pHYD6523. Using plasmid pHYD6521 as template the region between the XhoI and HindIII sites, was subjected to error prone PCR (63) with the primer pair JGKYCGOS2 and PTRCRP. The PCR amplicon was digested with XhoI and HindIII and ligated to XhoI and HindIII digested pHYD6523, the *kan* element serving as a stuffer. A pooled plasmid preparation from the obtained recombinants was then transformed into the strain JD509 (∆*ptsN* ∆*ycgO*::*kan*) and transformants were obtained on LB agar containing ampicillin. Multiple transformants were tested for the alleviation of the K^L^ owing to inactivation of YcgO_NFX_, on K_115_ agar containing 10 μM IPTG. Following evaluation of the spectrum of missense mutations amongst these transformants, site directed mutagenesis was employed to ascertain causation of a given missense mutation in alleviation of the K^L^. Mutant proteins that were expressed to a level equivalent to YcgO_NFX_ were chosen. The corresponding plasmids were transformed into the strain GJ22206 and the ability of the mutant *ycgO* genes to alleviate the K^L^ of GJ22206 was assessed and is displayed in Fig. 3A. The medium for all genetic procedures involving the strain GJ22206 is K_1_ medium.

**Immunoblotting and preparation of cell extracts for immunoblotting.**

Proteins separated by SDS PAGE were transferred to a PVDF membrane. Blocking of the PVDF membrane was performed with Tris-buffered saline (pH-7.4) containing 0.1% tween-20 (TBST buffer) and 5% skimmed milk for 30 minutes and the membrane was probed with either monoclonal anti-FLAG M2 antibody (F3165) or anti-6X His (anti-His) tag antibody (ab9108) at 4°C, overnight at a dilution of 1:10000 in TBST buffer, washed three times with 10 ml TBST buffer. The PVDF membrane was then probed with horseradish peroxidase (HRP)-conjugated secondary antibody (anti-Mouse [A4416] for anti-FLAG and anti-Rabbit [4010-05] for anti-His) for 2 hours at a dilution of 1:10000 in TBST buffer at room temperature and then washed thrice with the same buffer. Western blot was developed with the ECL solution kit and visualized in an ImageQuant LAS500 Imaging system. Representative images of western blots performed in duplicates are shown. For immunoblotting experiments, strains expressing various 3x FLAG tagged YcgO proteins, were inoculated in appropriate media and cultivated to an *A_600_* of 0.4-0.8. Cells normalized to an *A_600_* of 1, were pelleted and resuspended in 100 µl of buffer X (65 mM Tris-HCl pH 6.8 and 5% SDS). Samples were briefly sonicated, 100 µl of 2x SDS Laemmli buffer was added and 20-30 µl of samples were electrophoresed on 12% SDS PAGE gels.

**immunoblot processing.**

**Figure 3C:** Horizontally cropped images of a full gel following probing of the PVDF membrane with anti-FLAG antibody and staining of the same membrane with amido- black, are displayed for panels I and II respectively.

**Figure 4:** Input and elution fractions were loaded on one gel, following electrophoresis and transferred to a PVDF membrane, the membrane was cut horizontally below the 33 kDa marker. The top and the bottom portions were probed separately with anti-FLAG and anti-His antibodies separately. The top and the bottom portions were cut to separate the input and the elution samples that were developed separately. Cropped images of each portion are displayed.

**Figure 5A:** Immunoblot processing and image acquisition is as per figure 4.

**Figure 5B:** Input and elution fractions were loaded on two separate gels, following electrophoresis and transferred to a PVDF membrane, the membrane was cut horizontally below the 33 kDa marker. The top and the bottom portions of the membrane were probed separately with anti-FLAG and anti-His antibodies and developed separately. Cropped images of each portion are displayed.

**Figures S2 and 1C:** Vertically and horizontally cropped images of full gels following probing of the PVDF membrane with anti-FLAG antibody are displayed.

**Figure S4A and B:** Immunoblot processing and image acquisition is as per figure 3C.

**Figure S9:** Input and elution fractions were loaded on a pair of gels. Identical order of loading was performed. Following electrophoresis and transfer, the two PVDF membranes, were cut vertically to separate the input and elution fractions, and a pair derived from one gel was probed with anti-FLAG antibody and the other pair was probed with anti-His antibody.

**Figure S10:** Immunoblot processing and image acquisition is as per figure 4.

**Figure S12:** Immunoblot processing and image acquisition is as per figure 4.

**Protein purifications**

**A. Purification of unphospho-PtsN_His_**

For purification of C-terminally hexahistidine tagged unphospho-PtsN (unphospho-PtsN_His_), the strain GJ21015 bearing the plasmid pHYD5006 was cultured in LB broth with appropriate antibiotic selection at 37˚C, till mid-log phase (*A_600_* 0.4-0.5). IPTG (at 1 mM) was added to the culture to induce the expression of C-terminally hexahistidine tagged PtsN_His_, from the P*_trc_* promoter of pHYD5006. Glucose at 0.2% was also added and the culture was incubated further for 2.5 hours. Cells were pelleted and washed with buffer A (20 mM Tris-HCl, pH 8.0, 200 mM NaCl, 10% glycerol and 5 mM β-mercaptoethanol [BME]), suspended in lysis buffer (buffer A containing 1mg/ml lysozyme and 1 mM phenylmethylsulfonyl fluoride [PMSF]). Cell lysis was achieved using a One Shot cell disrupter (Constant Systems), and cell debris was removed by centrifugation at 18000 rpm/30 minutes at 4˚C. The supernatant was transferred into a Falcon tube, and 10 mM imidazole and Ni-NTA beads, washed and pre-equilibrated in buffer A containing 10 mM imidazole, were added to the lysate and kept at 4˚C for 3 hours with rotary mixing. Following transfer to a gravity column, the Ni-NTA beads were washed with 50 bead volumes of buffer A containing 25 mM imidazole, and elution of PtsN_His_ was performed in buffer A containing 300 mM imidazole. Unphospho-PtsN_His_ was dialyzed in buffer A and concentrated using an Amicon centrifugal filter column (10 kDa cut off).

**B. Purification of phospho-PtsN_His_**

For the purification of phospho-PtsN_His_, the strain GJ21016 that lacks PtsN and SixA, was co-transformed with two plasmids namely pHYD6580 and pHYD6581. pHD6580 is capable of overexpressing PtsN_His_ from the IPTG inducible P*_trc_* promoter whereas pHYD6581 is capable of overexpressing PtsP and PtsO from two separate T7 promoters. GJ21016, in addition, bears a chromosomal copy of the gene encoding T7 RNA polymerase, placed under the expression control of osmotically inducible *proU* promoter, P*_proUT7RNAP_* (68) permitting its induction by addition of NaCl, leading to the overproduction of PtsP and PtsO from the plasmid pHYD6581. An early exponential phase culture of GJ21016 cultivated in LB broth, bearing pHYD6580 and pHY6581 was generated and IPTG (at 100 μM) and NaCl (at 0.4 M) were added to this culture and the culture was incubated further for 3.5 hours. PtsN_His_ was subsequently purified via Ni-NTA affinity chromatography as described above. The purified protein was dialyzed in buffer B (20 mM Tris-Cl pH 8.0, 50 mM NaCl, 5 mM BME and 5% glycerol), concentrated in the same buffer and injected into a 1 ml Hi Trap ^TM^ QFF anion exchange column pre-equilibrated with buffer B lacking glycerol, in the Äkta Purifier system at a flow rate of 0.2 ml per minute. PtsN_His_ was eluted in a 0.05 to 0.5 M NaCl gradient. Multiple fractions were collected throughout the elution trace, and aliquots were electrophoresed on a 15% native PAGE, under the conditions described in reference 23, to detect fractions enriched in either phospho or unphospho-PtsN. Two species of PtsN were detected with fractions displaying a distribution in the proportion of the two species. The slow and the fast-migrating species represent respectively unphospho and phospho-PtsN (39, 40). Fractions marked in the trace (Fig. S11A) as 1 and 4 corresponding to unphospho and phospho-PtsN respectively (Fig. S11B), were collected and buffer exchanged into buffer A using a 10 kDa cut off Amicon centrifugal filter column.

**C. Purification of NusG_His_**

The protocol for purification of C-terminally hexahistidine tagged NusG (NusG_His_) was identical to the protocol for purification of unphospho-PtsN_His_, except that expression of NusG_His_ from the T7 promoter of the plasmid pRS119 (42) was induced by addition of IPTG (at 1 mM) to a mid-log phase culture of BL21 [DE3] (57), that was cultivated further for 3 hours prior to preparation of cell extract for protein purification.

**D. Purification of unphospho-PtsN_His_ for fluorescence-detection size exclusion chromatography (FSEC)**

Following overproduction of PtsN_His_ with 1 mM IPTG, from the plasmid pHYD6580 in the strain GJ21015 as described in protocol 1, cells were pelleted at 4000 *g* at 4°C for 20 minutes, and resuspended in HEPES buffered saline (HBS, 20 mM HEPES pH 7.0, 150 mM NaCl) containing 1 mM BME and 10% (v/v) glycerol (PtsN purification buffer). Cells were lysed through high pressure homogenization at 700-800 bar pressure, and the lysed cell suspension was clarified by ultracentrifugation at 111000 *g* for 30 minutes at 4°C. The supernatant was mixed with Ni-NTA beads pre-equilibrated with PtsN purification buffer and 10 mM imidazole, and kept on end-on nutation for 2 hours at 4°C. The suspension was transferred to a gravity column, where the beads were washed with 30 bead volumes of 30 mM imidazole in PtsN purification buffer, and unphospho-PtsN_His_ was eluted in 5 bead volumes (~10 ml) of PtsN purification buffer containing 300 mM imidazole. The protein was concentrated to a final volume of 0.5 ml before injecting it into GE Superdex 75 10/300 GL column for size exclusion chromatography, with 20 mM HEPES pH 7.0 and 200 mM NaCl kept in the mobile phase. Due to the absence of tryptophan residues in PtsN_His_, the protein concentration was estimated using the Bradford method.

**E. Purification of unphospho-PtsN_His_ and a preparation of PtsN_His_ containing a mixture of unphospho and phospho-PtsN_His_ for LC ESI-MS**

For purification of unphospho-PtsN_His_, the strain GJ19300 bearing the plasmid pHYD5006 was cultured in LB broth containing appropriate antibiotic at 37˚C, till the mid-log phase (*A_600_* 0.4-0.5). Expression of PtsN_His_, was induced with the addition of IPTG (at 1 mM) and glucose (at 0.2%) was added to the culture that was cultivated further for 2 hours. Cells were pelleted and washed with buffer C (20 mM Tris-HCl, pH 7.5, 50 mM NaCl, and 10% glycerol). All other steps were identical to those followed for the purification of unphospho-PtsN_His_ described above except that buffer C was used throughout the purification protocol.

For the purification of a PtsN_His_ preparation containing a mixture of phospho and unphospho forms, the strain GJ21012, was co-transformed with two plasmids namely pHYD6542 and pHYD5006. In pHYD6542, PtsP and PtsO can be expressed from a pair of T7 promoters and pHYD5006 has been described above. The strategy to co-express PtsN_His_, PtsP and PtsO by the addition of IPTG and NaCl, described above was employed. All other steps remained identical to those followed for purification of unphospho-PtsN_His_ with buffer C being used throughout. Protein samples were dialyzed and concentrated in a buffer containing 10 mM Tris-HCl, pH 7.5, 50 mM NaCl, and 5% glycerol. Preparation and processing of the protein samples for LC ESI-MS described in LC ESI-MS of PtsN section.

**F.** **Purification of YcgO_FH_**

C41 [DE3] (58) cells bearing the plasmid pHYD5011 expressing YcgO_FH_, that is YcgO bearing a 3x FLAG and a 6x His tag appended to its carboxyl-terminus (immediately after the penultimate amino acid) were cultivated at 37°C, to an *A_600_* of 0.4-0.5, at which point the expression of YcgO_FH_ was induced by addition of 1 mM IPTG and the culture was incubated at 18°C overnight. Cells were pelleted at 4000 *g* for 20 minutes at 4°C, and resuspended in potassium-HEPES buffer (K-HBS, 20 mM HEPES, pH 7.0 and 200 mM KCl) with 1 mM PMSF and 5% (v/v) glycerol. Cells were lysed using GEA Niro Soavi PandaPlus high pressure homogenizer over 3 to 4 passes at 700-800 bar pressure. The suspension was ultracentrifuged at 111000 *g* at 4°C for 90 minutes to isolate membranes. The membranes were resuspended in ~40 ml resuspension buffer containing K-HBS, 1 mM PMSF and 5% glycerol, and a final concentration of 20 mM lauryl maltoside neopentyl glycol (LMNG) was added to the suspension for solubilizing YcgO_FH_. The suspension was kept on end-on nutation for 5 hours, after which undissolved membranes were removed by ultracentrifugation at 111000 *g* at 4°C for 60 minutes. Imidazole at 10 mM, was added to the supernatant which was then mixed with Ni-NTA beads that were pre-equilibrated with K-HBS, 5% (v/v) glycerol, 1 mM PMSF, and 0.1 mM LMNG (YcgO purification buffer), and kept on end-on nutation for 3 to 4 hours at 4°C. The beads were transferred into a gravity column and washed with 40 column volumes of YcgO purification buffer containing 30 mM imidazole. YcgO_FH_ was eluted in 5 column volumes (~10ml) of 300 mM imidazole in YcgO purification buffer. The eluate was concentrated to 0.5 ml and passed through a GE Superose 6 increase 10/300 GL column for size exclusion chromatography with K-HBS and 0.1 mM LMNG in the mobile phase, where YcgO_FH_ eluted at ~15.5 ml volume. Protein concentration was estimated through normalizing UV absorbance at 280 nm with the extinction coefficient of the protein as predicted by Protparam server (<https://web.expasy.org/protparam>).

**G**. **CTR_His_ purification**

For purification of CTR_His_, pHYD6547, that encodes the C-terminally hexahistidine tagged CTR of YcgO spanning amino acids from 386 to 578, was transformed into the strain BL21[DE3] (57). BL21 [DE3] bearing the plasmid pHYD6547 was cultivated at 37°C till mid-exponential phase in LB broth with the appropriate antibiotic. Expression of CTR_His_ was induced by the addition of IPTG (at 1 mM) to the culture that was further incubated for 4 hours. Cells were pelleted and washed with buffer D (20 mM Tris-HCl pH 6.8, 200 mM NaCl, 10% glycerol and 5 mM BME). The pellet was resuspended in buffer D containing 1 mg/ml lysozyme and 1 mM PMSF. The procedure for cell lysis and Ni-NTA purification was as per protocol 1, except that buffer D was used for binding of CTR_His_ to the Ni-NTA beads. Whereas washes and elution were performed in buffer E (20 mM sodium phosphate pH 6.8, 200 mM sodium fluoride (NaF), 10% glycerol, 5 mM BME) containing 25 mM and 300 mM imidazole respectively. Purified CTR_His_ was dialyzed into buffer F (20 mM sodium phosphate pH 6.8, 200 mM sodium fluoride and 0.1 mM BME) and concentrated using an Amicon centrifugal filter.

**Circular dichroism (CD) analysis of CTR_His_**

Circular dichroism spectroscopy of purified CTR_His_ was performed using a JASCO (J-810) spectropolarimeter, at room temperature, in buffer F. The CD spectrum was obtained in the 260 to 190 nm range with the protein solution in a cuvette with a path length of 0.1 cm. CTR_His_ was present at 0.22 mg/ml. The following settings were used for the measurements: 1 nm data pitch, 2 nm bandwidth, 4 seconds response time, and 100 nm/minute scanning speed. The estimations of secondary structures were performed as described in reference 69.

**LC ESI-MS of PtsN**

To preserve histidine phosphorylation in PtsN protein preparations purified for LC ESI-MS, the protein was precipitated out of the solution using methanol-chloroform extraction, where the protein sample (100 μl) was taken and 200 μl methanol was added and mixed, followed by addition of 100 μl chloroform. 200 μl of deionized water was added to this solution and mixed vigorously through vortexing. The suspension was centrifuged at 10000 *g* for 10 minutes at room temperature to assist in phase separation, with the protein precipitating at the interface of the two phases. The top phase was aspirated, and to the remnant solution and precipitant, 300 μl methanol was added. The precipitant was flicked off gently and the sample was centrifuged again at 10000 *g* for 2 minutes to settle the precipitate at the bottom of the microcentrifuge tube. The liquid was decanted and remnant liquid in the microcentrifuge tube was air-dried. The tube with the dried precipitated sample was stored at -80°C, and resuspended in 60% (v/v) formic acid immediately prior to LC ESI-MS. The same procedure was followed for processing unphospho- PtsN_His_.

For mass spectrometry, the sample was subjected to RP-HPLC through HPLC 1260 Infinity (Agilent technologies) coupled with Maxis Impact ESI-MS (Bruker Daltonics, Bremen, Germany). 100 μl of sample was injected in C8 Zorbax 300SB, 3.1x100 mm RP column. The mobile phase consisted of a constant 0.1% formic acid and a gradient of 10% to 90% acetonitrile in water. Populations were quantified for the elution volume ranging between 11.7-13.1 minute in the 25 minute LC runs, as majority of the PtsN eluted in this volume. Within this range, spans where isolated phosphorylated and unphosphorylated populations were predominant were integrated and m/z series graphs plotted in Figure S8A and B respectively. For both unphosphorylated and phosphorylated PtsN_His_ protein samples, the mass spectrometry was performed in two biological replicates.

**Preparation of everted vesicles**

Everted vesicles were prepared from the strain GJ22224 with the described plasmids. GJ22224 bearing pHYD6578, that is capable of expressing C-terminally 3x FLAG tagged YcgO, from the L-arabinose (Ara) inducible P*_ara_* promoter of the plasmid pBAD24 (46) and the vector pBAD24, were cultivated in KML broth (66) supplemented with 0.2% glucose and the appropriate antibiotic. Following overnight growth, the two cultures were sub-cultured into KML broth and cultivated to an *A_600_* of 0.6. Ara at 0.05% was added to both cultures that were further incubated at 20°C for 8 hours. Culture pellets were washed thrice with buffer A (140 mM choline chloride, 10% (v/v) glycerol, 20 mM Tris-HCl pH 7.5) and then resuspended in 50 to 60 ml of buffer A containing 1 mM DTT, 0.1 mM PMSF and 5 mg/L DNase I. The suspension was homogenized at 400 bar pressure for 90 seconds, and then ultracentrifuged at 111000 *g* for 120 minutes. The pellet was resuspended in buffer A containing 1 mM DTT, 0.1 mM PMSF and 1 mM MgCl_2_, aliquoted in 30 μl volumes, and snap frozen in liquid nitrogen for storage and later use.

**Figure S1**

**
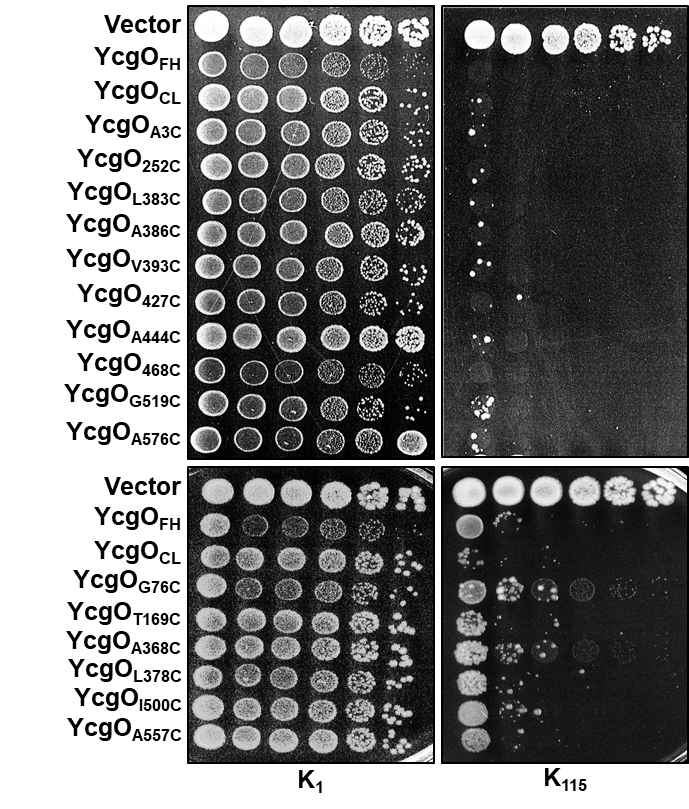
**

**Figure S1.** The functionality of 3x FLAG 6x His-tagged YcgO (YcgO_FH_), its Cysless derivative (YcgO_CL_) and the indicated mono-Cys versions. Ten- fold serial dilutions of cultures of the ∆*ptsN* ∆*ycgO*::*kan* double mutant JD509 bearing the vector pTrc99A, expressing at the basal level, the indicated YcgO proteins, were spotted on the surface of glucose minimal agar plates with K^+^ content of 1 mM (K_1_) and 115 mM (K_115_). The mono-Cys substitution is indicated in subscripted lettering.

S-29

**Figure S2**

**
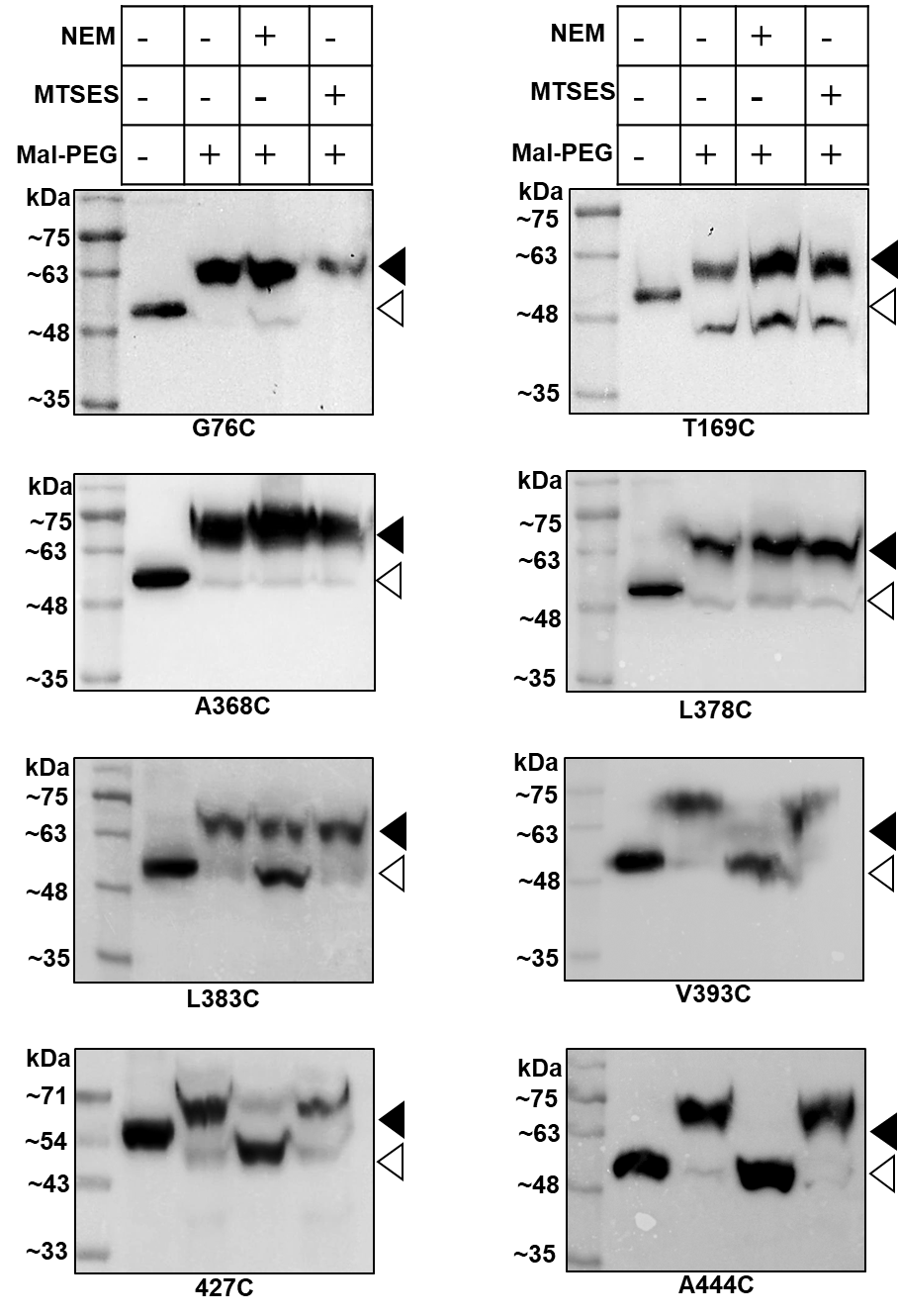
**

S-29

**Figure S2 (Contd.)**

**
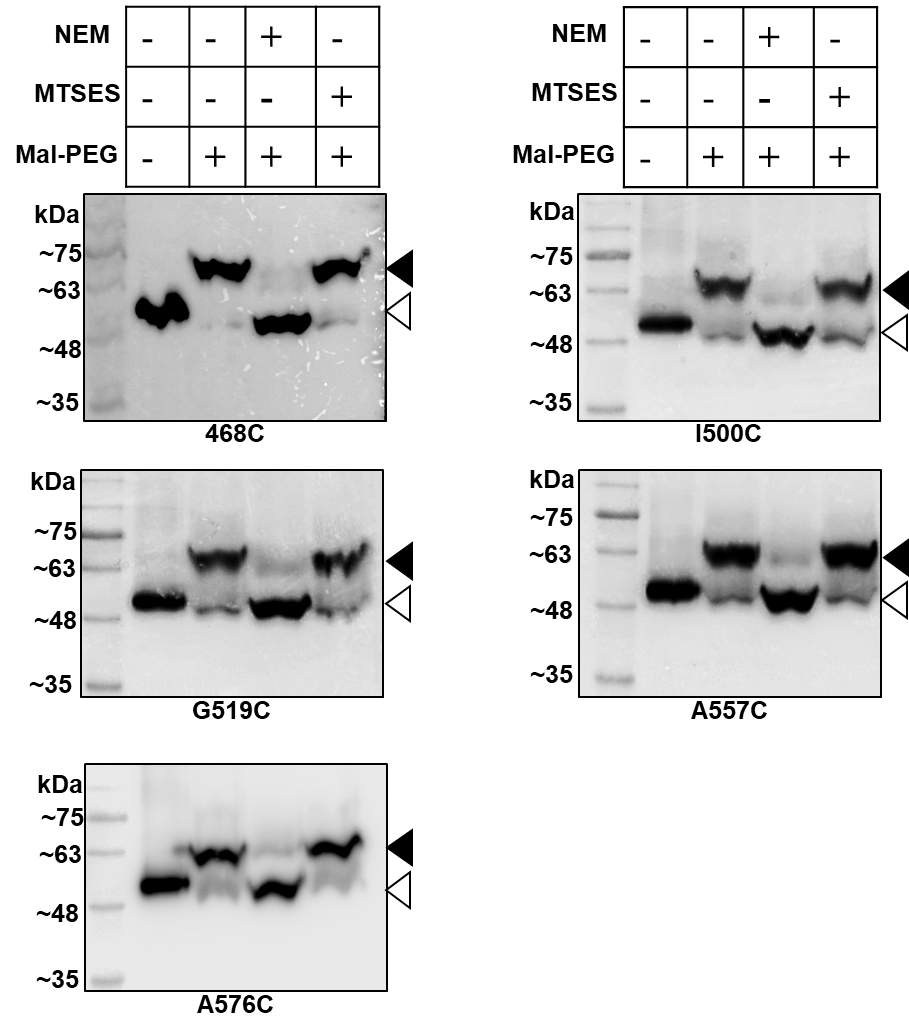
**

**Figure S2.** Anti-FLAG immunoblots depicting accessibility of the indicated mono- Cys substituted derivatives of the Cysless YcgO (YcgO_CL_) to NEM and MTSES. Mid-log phase cultures of the strain GJ18836 bearing plasmids expressing the indicated mono-Cys of YcgO_CL_, were harvested and processed as described in experimental procedures. Treatments with NEM, MTSES and Mal-PEG are indicated. Open and filled triangles indicate respectively the positions of the free and the YcgO:Mal-PEG adducts. All YcgO proteins are expressed from the plasmid-borne P*trc* promoter and the corresponding plasmids are listed in Table S2.

S-30

**Figure S3**

10

Helix: 7.10 ± 3.82%

β-sheet: 53.45 ± 10.82%

Turn: 0%

Random: 39.5 ± 6.93%

0

**Molar ellipticity**

-10

-20

-30

-40

190

200 220 240

**Wavelength (nm)**

260

**Figure S3**. Circular dichroism spectrum of purified YcgO CTR_His_. Percentage of secondary structure elements (boxed) are indicated. The spectrum was recorded with CTR_His_ present at 0.22 mg/ml in a buffer comprising 20 mM sodium phosphate pH 6.8, 200 mM sodium fluoride and 0.1 mM β- mercaptoethanol. Two blank subtracted spectra were recorded with the same protein preparation.

S-31

**Figure S4**

**
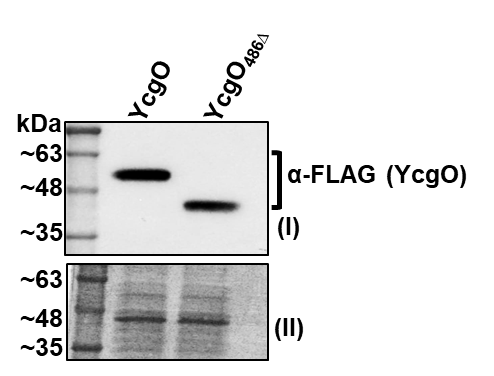

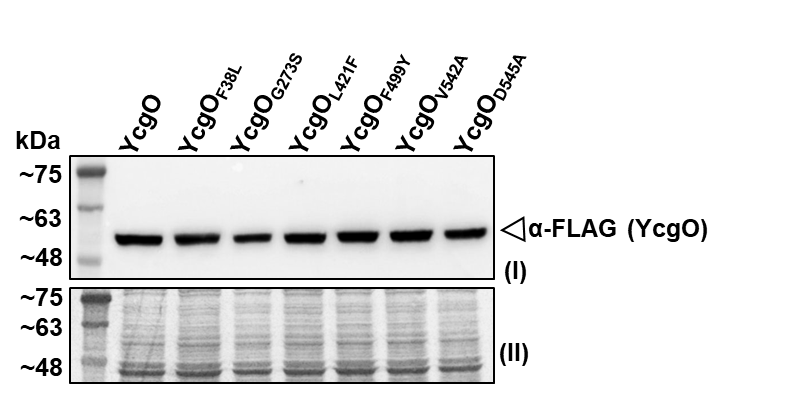
**

**B**

**A**

**Figure S4.** Expression levels of YcgO^Con^ phenotype conferring proteins. (A) Cultures of the strain GJ17854 that expresses from the chromosome C- terminally 3x FLAG tagged YcgO (YcgO) and its indicated substitution derivatives were cultivated till the mid-exponential phase in K_1_ medium. Cell extracts prepared from *A_600_* normalized cultures were electrophoresed on SDS- PAGE, transferred to a PVDF membrane and probed with anti-FLAG antibody (I). A portion of the membrane stained with amido-black (II) as loading control is indicated. Panel B represents anti-FLAG immunoblot displaying the expression level of YcgO and YcgO_486∆_. Strains used in panels A are GJ17854, GJ19263, GJ19264, GJ19265, GJ19266, GJ19267, GJ19290 and in panel B are GJ17854 and GJ19291.

S-32

**Figure S5**

**
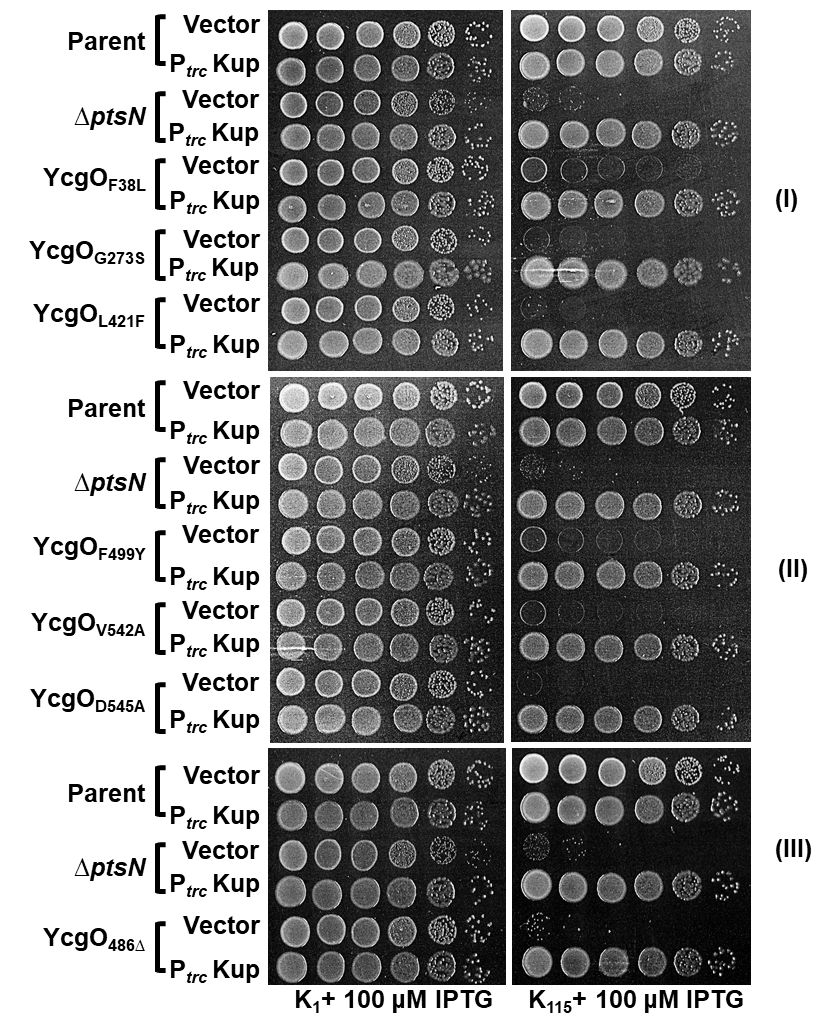
**

**Figure S5.** Alleviation of the YcgO^Con^ phenotype by overexpression of Kup. Strains with the chromosomal expression of C-terminally 3x FLAG tagged YcgO (parent, ∆*ptsN*) and the parent expressing the indicated YcgO proteins in panels I, II and III were transformed with the vector (pHYD5001) and the plasmid pHYD1852 (P*_trc_*Kup) and their cultures were spotted on K_1_ and K_115_ agar plates containing 100 μM IPTG. Strains used in panel I are GJ19267, GJ19290 and GJ19264; in panel II are GJ19263, GJ19265 and GJ19266; the strain in panel III is GJ19291. GJ17854 (parent) and GJ22207 (*∆ptsN*) are common to all panels.

S-33

**
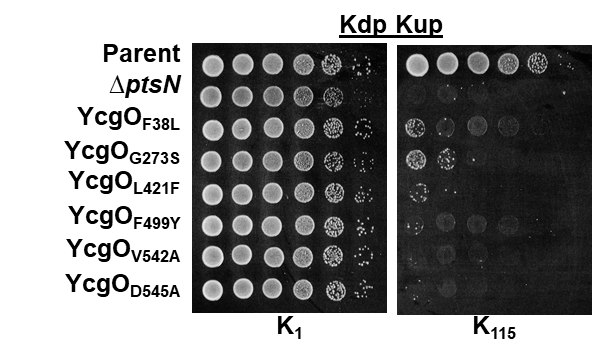

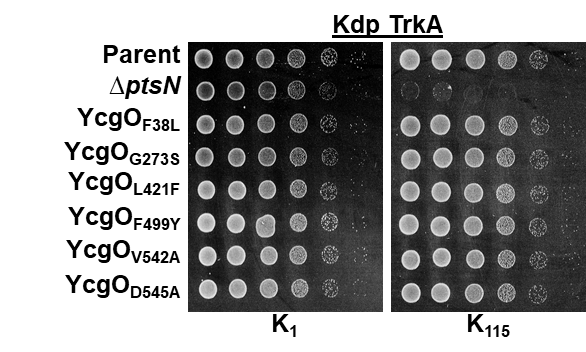

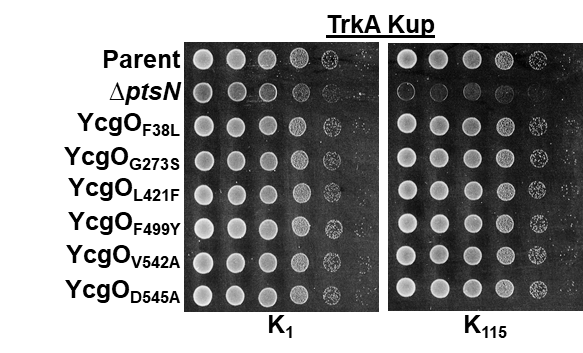
**

**Figure S6**

**C**

**B**

**A**

**Figure S6.** Expression of the YcgO_Con_ phenotype requires the absence of the Trk K^+^ uptake system. Serial dilutions of cultures of the parent and its ∆*ptsN* derivative expressing C-terminally 3x FLAG tagged YcgO and the parent expressing the indicated YcgO^Con^ phenotype conferring substitution derivatives were spotted on the surface of K_1_ and K_115_ agar plates. The active K^+^ uptake systems present in the group of strains in panels A, B and C are indicated. Strains used in panels A, B and C are marked with superscripted numbers 1, 2 and 3 respectively in Table S1.

S-34

**C**

**Figure S7**

**
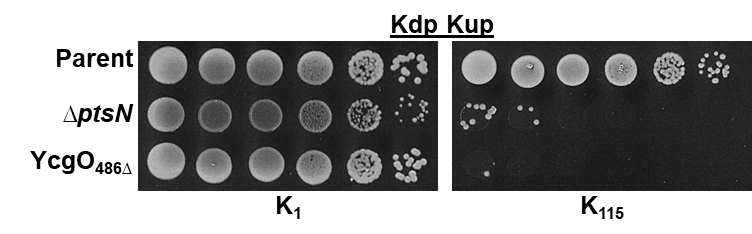

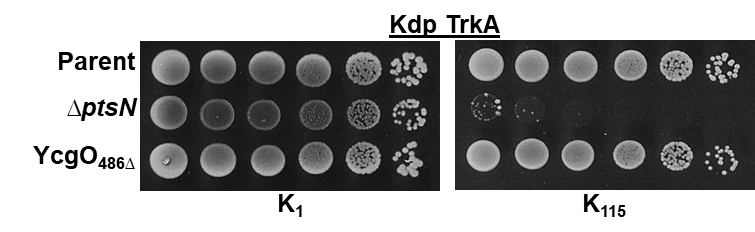

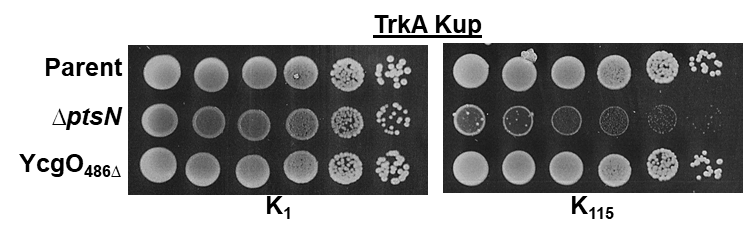
**

**B**

**C**

**A**

**Figure S7**. The YcgO^Con^ phenotype conferred by chromosomal expression of YcgO_486∆_ requires the absence of the Trk K^+^ uptake system. Serial dilutions of cultures of the parent and its ∆*ptsN* derivative expressing C-terminally 3x FLAG tagged YcgO and the parent expressing YcgO_486∆_ were spotted on the surface of K_1_ and K_115_ agar plates. The active K^+^ uptake systems present in the group of strains in panels A, B and C are indicated. Strains used in panels A, B and C are marked with superscripted numbers 4, 5 and 6 respectively in Table S1.

S-35

**Figure S8**

**A** Intens. x105

+MS, 11.8-12.7min #704-763, Smoothed (0.14,1,SG)

22+

854.6929

20+

940.0610 19+

989.4837

18+

1044.3868

23+

81 60

7.57

17+

1105.7577

16+

1174.8517

15+

1253.1100

14+

1342.5474

13+

14 53

45.74

12+

1566.1358

11+

1708.4100

10+

1879.1463

1392.2441

1503.5503

1634.2121

1789.7673

| **Mass** | **Abundance** | **Abundance(%)** | **Std. dev.** |
| --- | --- | --- | --- |
| **18780.67** | **1348110** | **100** | **0.293419** |
| **18649.19** | **281558** | **20.89** | **0.239779** |
| **19758.10** | **47289** | **3.51** | **0.28862** |

1.0

0.8

0.6

0.4

0.2

0.0

# B


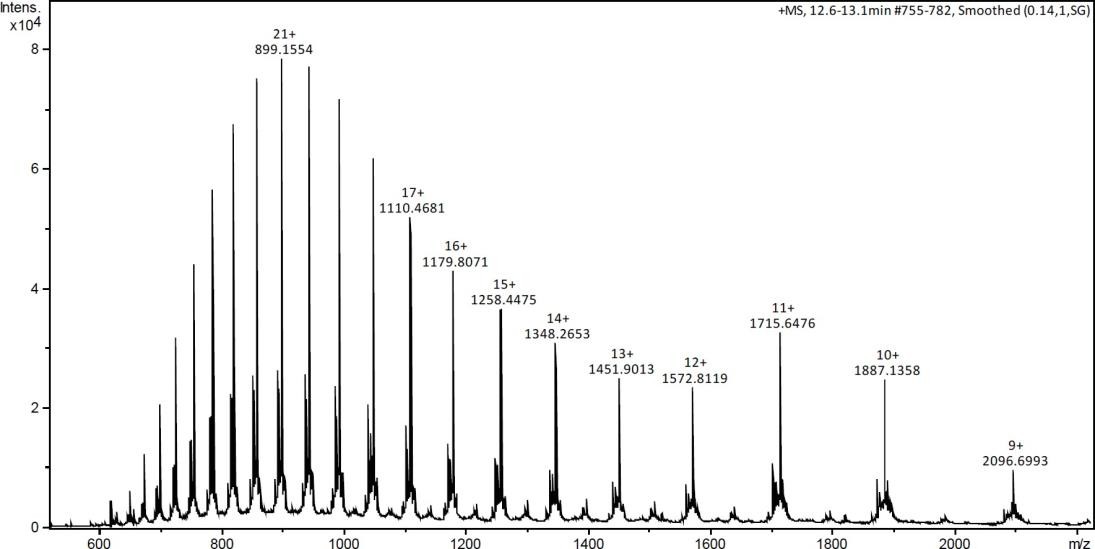


800 1000 1200 1400 1600 1800 m/z

| **Mass** | **Abundance** | **Abundance(%)** | **Std. dev.** |
| --- | --- | --- | --- |
| **18860.52** | **891430** | **100** | **0.325637** |
| **18729.17** | **282963** | **31.74** | **0.248033** |
| **18777.27** | **255394** | **28.65** | **0.23031** |

**Figure S8: Mass spectrometry-based quantification of histidine phosphorylation states in purified PtsN.** Abundance in percentage are normalized to the most abundant species detected in the spectra, and not against the total detection above a fixed threshold. **(**A) LC ESI-MS spectrum of unphospho-PtsN_His_ protein preparation. Only pure unphospho-PtsN_His_ (molecular mass of 18781 Da) could be detected. 18649.19 Da corresponds to the molecular mass of unphospho-PtsN_His_ with first methionine cleaved, while a minor population with a mass of 19758.10 Da could not be attributed to any modification to unphospho-PtsN_His_. (B) LC ESI-MS spectrum of PtsN_His_ purification that yielded a mixture of phospho-PtsN_His_, including 18860 Da species that represents PtsN_His_ with one histidine phosphorylated, and 18729 Da species that corresponds to the 18860 Da species with the N-terminal methionine cleaved. The mass of 18777.27 Da could plausibly represent unphospho-PtsN_His_.

S-36

**Figure S9**

**
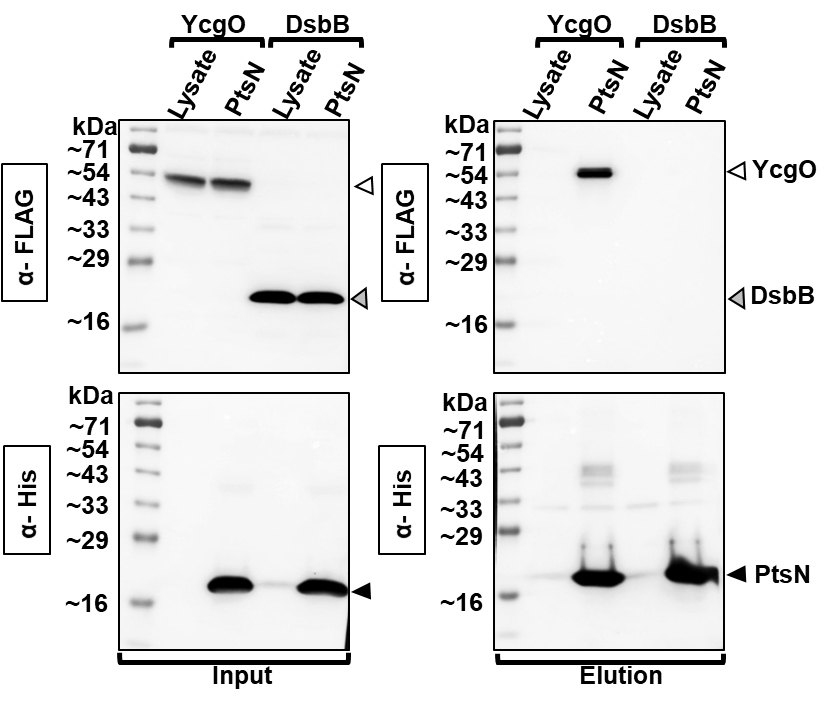
**

**Figure S9.** Specific interaction of unphospho-PtsN with YcgO. Immunoblots depicting the outcome of co-purification of 3x FLAG tagged YcgO (YcgO) and 3x FLAG tagged DsbB (DsbB) with unphospho-PtsN_His_ (PtsN). Co-purifications were performed as described in the experimental procedures section (and Fig. 4). *A_600_* normalized volumes of DDM solubilized cell extracts of the strain GJ21020 and GJ21019 expressing respectively C-terminally 3x FLAG tagged YcgO and DsbB were used to test their ability to co-purify with unphospho-PtsN_His_. Lanes marked “Lysate” indicate that lysates of GJ21020 and GJ21019 without exposure to unphospho-PtsN_His_, were loaded and in lanes marked “PtsN”, purified unphospho-PtsN_His_ was mixed with the aforementioned lysates. Anti-FLAG and anti-His immunoblots for detection of YcgO, DsbB and PtsN proteins respectively are marked with rectangular boxes. The positions of YcgO, DsbB and unphospho-PtsN_His_ are marked with white, light grey and black triangles respectively. Immunoblot processing for this figure is described in the methods section of supporting information.

S-37

**
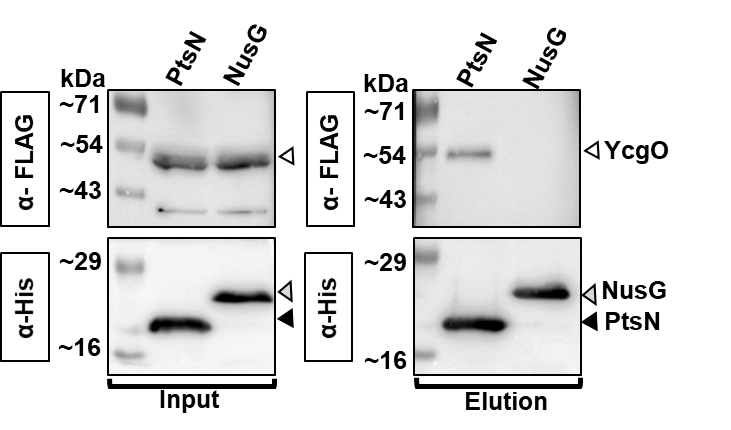
**

**Figure S10**

**Figure S10.** Absence of co-purification of YcgO with 6x His tagged NusG. Immunoblots depicting the interaction of 3x FLAG tagged YcgO (YcgO) with unphospho-PtsN_His_ (PtsN) and the absence of interaction with NusG_His_ (NusG). Co-purification was performed as described in the experimental procedures section. All procedures were as per Figure 4. Anti-FLAG and anti-His immunoblots for detection of YcgO and PtsN proteins respectively are marked with rectangular boxes. The positions of YcgO, NusG_His_ and unphospho-PtsN_His_ are indicated with white, light grey and black triangles respectively.

S-38

**Figure S11**

#
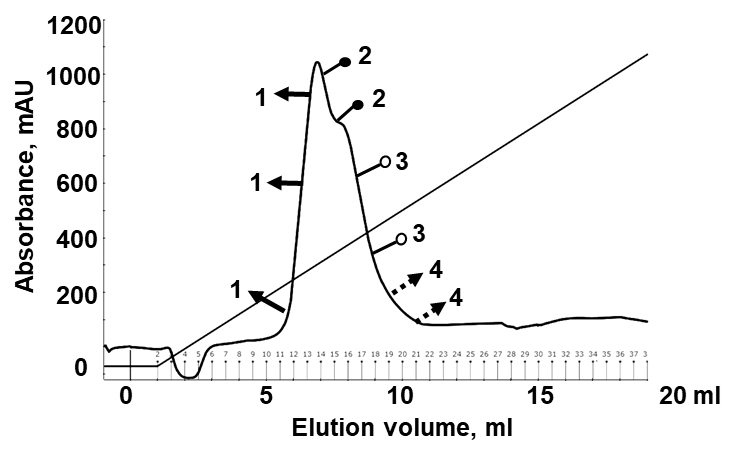


**A**

#
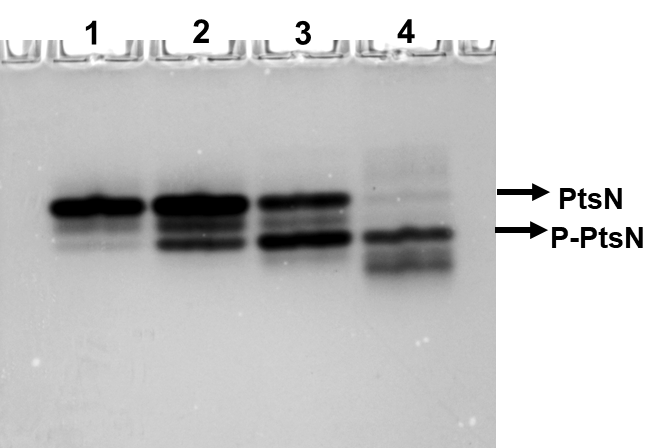


**B**

**Figure S11.** Separation and purification of unphospho-PtsN_His_ (PtsN) and phospho-PtsN_His_ (P-PtsN). Buffer exchanged, Ni-NTA purified preparation of PtsN_His_ purified as described under “Purification of phospho-PtsN_His_” was loaded onto a 1 ml Hi Trap^TM^ QFF anion exchange column pre-equilibrated with appropriate buffer using an Äkta Purifier. Following chromatography samples were withdrawn from marked fractions corresponding to the chromatograph trace (A), pooled and electrophoresed on a 15% native polyacrylamide gel that was stained with Coomassie brilliant blue (B). Fractions marked 1 and 4 corresponding to unphospho-PtsN_His_ and phospho-PtsN_His_ respectively, were chosen to perform co-purification with YcgO (Figure 4).

S-39

**Figure S12**

**s**

**
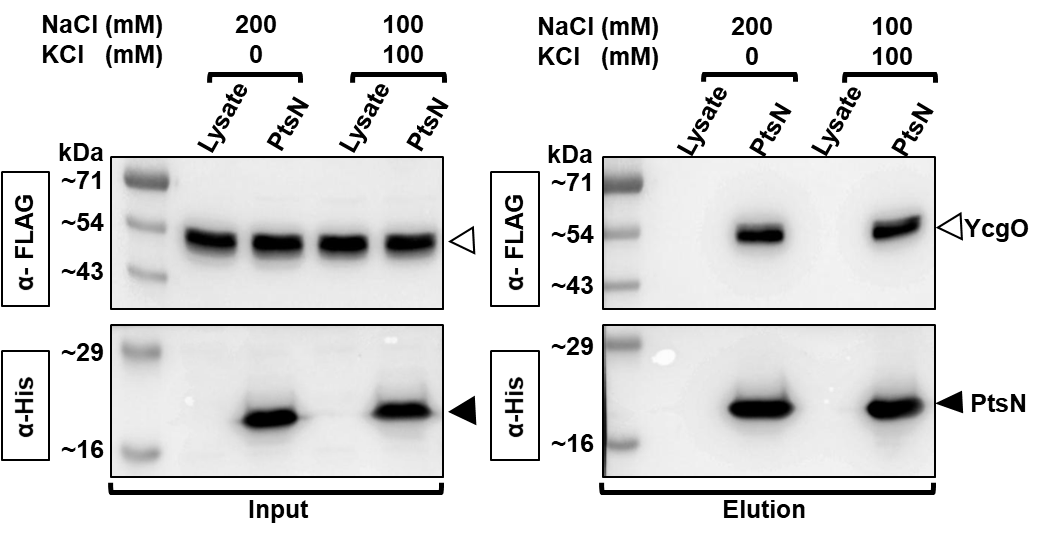
**

**Figure S12.** Co-purification of YcgO with unphospho-PtsN_His_ in a buffer containing NaCl and KCl each at 100 mM. Co-purification of YcgO with unphospho-PtsN_His_ (PtsN) was tested in buffers with 200 mM NaCl and a mixture of 100 mM NaCl and 100 mM KCl. Co-purifications were performed as per Figure 4. Lanes marked “Lysate” indicate that lysates of GJ21020 without exposure to unphospho-PtsN_His_, was loaded and in lanes marked “PtsN”, purified unphospho-PtsN_His_ was mixed with the aforementioned lysates. The boxed α- FLAG and α-His labelling represents images of immunoblots probed with anti-FLAG and anti-His antibodies respectively. The positions of YcgO and unphospho-PtsN_His_ are marked with white and black triangles respectively. Unphospho-PtsN_His_ was purified in a buffer containing 100 mM NaCl.

**References**

15. Sharma, R., Shimada, T., Mishra, V. K., Upreti, S., and Sardesai, A. A. (2016) Growth inhibition by external potassium of *Escherichia coli* lacking PtsN (EIIA_Ntr_) is caused by potassium limitation mediated by YcgO. *J. Bacteriol.* **198**, 1868–1882.

23. Schulte, J. E., and Goulian, M. (2018) The phosphohistidine phosphatase SixA targets a phosphotransferase system. *mBio*. **9**, e01666-18.

37. Andrews, A. E., Lawley, B., and Pittard, A. J. (1991) Mutational analysis of repression and activation of the *tyrP* gene in *Escherichia coli*. *J. Bacteriol.* **173**, 5068–5078.

39. Bahr, T., Lüttmann, D., März, W., Rak, B., and Görke, B. (2011) Insight into bacterial phosphotransferase system-mediated signaling by interspecies transplantation of a transcriptional regulator. *J. Bacteriol.* **193,** 2013–2026.

40. Lüttmann, D., Göpel, Y., and Görke, B. (2015) Cross-talk between the canonical and the nitrogen-related phosphotransferase systems modulates synthesis of the KdpFABC potassium transporter in *Escherichia coli*. *J. Mol. Microbiol. Biotechnol.* **25**, 168–177.

42. Cheeran, A., Babu Suganthan, R., Swapna, G., Bandey, I., Achary, M. S., Nagarajaram, H. A., and Sen, R. (2005) *Escherichia coli* RNA polymerase mutations located near the upstream edge of an RNA:DNA hybrid and the beginning of the RNA-exit channel are defective for transcription antitermination by the N protein from lambdoid phage H-19B. *J. Mol. Biol.* **352**, 28–43.

46. Guzman, L. M., Belin, D., Carson, M. J., and Beckwith, J. (1995) Tight regulation, modulation, and high-level expression by vectors containing the arabinose PBAD promoter. *J. Bacteriol.* **177**, 4121–4130.

57. Studier, F. W., and Moffatt, B. A. (1986) Use of bacteriophage T7 RNA polymerase to direct selective high-level expression of cloned genes. J Mol. Biol. **189**, 113–130.

58. Miroux, B., and Walker, J. E. (1996) Over-production of proteins in *Escherichia coli*: mutant hosts that allow synthesis of some membrane proteins and globular proteins at high levels. *J. Mol. Biol.* **260**, 289–298.

59. Miller, J. H. (1992) A Short Course in Bacterial Genetics, Cold Spring Harbor Laboratory, Plainview, NY.

60. Datsenko, K. A., and Wanner, B. L. (2000) One-step inactivation of chromosomal genes in *Escherichia coli* K-12 using PCR products. *Proc. Natl. Acad. Sci. U. S. A.* **97**, 6640–6645.

61. Uzzau, S., Figueroa-Bossi, N., Rubino, S., and Bossi, L. (2001) Epitope tagging of chromosomal genes in *Salmonella*. *Proc. Natl. Acad. Sci. U. S. A.* **98**, 15264–15269.

63. Pathania, A., Gupta, K., Dubey, S., Gopal, B., and Sardesai, A. (2016) The topology of the L-Arginine exporter ArgO conforms to an N _in_ -C _out_ configuration in *Escherichia coli*: requirement for the cytoplasmic N-Terminal domain, functional helical interactions, and an aspartate pair for ArgO function. *J. Bacteriol.* **198**, 3186–3199

64. Baba, T., Ara, T., Hasegawa, M., Takai, Y., Okumura, Y., Baba, M., Datsenko, K. A., Tomita, M., Wanner, B. L., and Mori, H. (2006) Construction of *Escherichia coli* K-12 in-frame, single-gene knockout mutants: the Keio collection. *Mol. Syst. Biol.* **2**, 2006.0008.

65. Amann, E., Ochs, B., and Abel, K. J. (1988) Tightly regulated *tac* promoter vectors useful for the expression of unfused and fused proteins in *Escherichia coli*. *Gene*. **69**, 301–15

66. Epstein, W., and Kim, B. S. (1971) Potassium transport loci in*Escherichia coli*K-12. J Bacteriol. **108**, 639–644.

68. Bhandari, P., and Gowrishankar, J. (1997) An *Escherichia coli* host strain useful for efficient overproduction of cloned gene products with NaCl as the inducer. *J. Bacteriol.* **179**, 4403–4406.

69. Yang, J. T., Wu, C. S., and Martinez, H. M. (1986) Calculation of protein conformation from circular dichroism. *Methods in enzymol.* **130,** 208–269.

70. Dubey, S., Majumder, P., Penmatsa, A., and Sardesai, A. A. (2021) Topological analyses of the L-lysine exporter LysO reveal a critical role for a conserved pair of intramembrane solvent-exposed acidic residues.  *J. Biol. Chem.*  **297**, 101168.
